# Multi-Scale Genomic, Transcriptomic and Proteomic Analysis of Colorectal Cancer Cell Lines to Identify Novel Biomarkers

**DOI:** 10.1371/journal.pone.0144708

**Published:** 2015-12-17

**Authors:** Romina Briffa, Inhwa Um, Dana Faratian, Ying Zhou, Arran K. Turnbull, Simon P. Langdon, David J. Harrison

**Affiliations:** 1 Division of Pathology, Institute of Genetics and Molecular Medicine, University of Edinburgh, Crewe Road South, Edinburgh, EH4 2XU, United Kingdom; 2 School of Medicine, University of St Andrews, St Andrews, KY16 9TF, United Kingdom; Sapporo Medical University, JAPAN

## Abstract

Selecting colorectal cancer (CRC) patients likely to respond to therapy remains a clinical challenge. The objectives of this study were to establish which genes were differentially expressed with respect to treatment sensitivity and relate this to copy number in a panel of 15 CRC cell lines. Copy number variations of the identified genes were assessed in a cohort of CRCs. IC_50_’s were measured for 5-fluorouracil, oxaliplatin, and BEZ-235, a PI3K/mTOR inhibitor. Cell lines were profiled using array comparative genomic hybridisation, Illumina gene expression analysis, reverse phase protein arrays, and targeted sequencing of *KRAS* hotspot mutations. Frequent gains were observed at 2p, 3q, 5p, 7p, 7q, 8q, 12p, 13q, 14q, and 17q and losses at 2q, 3p, 5q, 8p, 9p, 9q, 14q, 18q, and 20p. Frequently gained regions contained *EGFR*, *PIK3CA*, *MYC*, *SMO*, *TRIB1*, *FZD1*, and *BRCA2*, while frequently lost regions contained *FHIT* and *MACROD2*. *TRIB1* was selected for further study. Gene enrichment analysis showed that differentially expressed genes with respect to treatment response were involved in Wnt signalling, EGF receptor signalling, apoptosis, cell cycle, and angiogenesis. Stepwise integration of copy number and gene expression data yielded 47 candidate genes that were significantly correlated. *PDCD6* was differentially expressed in all three treatment responses. Tissue microarrays were constructed for a cohort of 118 CRC patients and *TRIB1* and *MYC* amplifications were measured using fluorescence *in situ* hybridisation. *TRIB1* and *MYC* were amplified in 14.5% and 7.4% of the cohort, respectively, and these amplifications were significantly correlated (p≤0.0001). *TRIB1* protein expression in the patient cohort was significantly correlated with pERK, Akt, and Caspase 3 expression. In conclusion, a set of candidate predictive biomarkers for 5-fluorouracil, oxaliplatin, and BEZ235 are described that warrant further study. Amplification of the putative oncogene *TRIB1* has been described for the first time in a cohort of CRC patients.

## Introduction

Colorectal cancer (CRC) accounts for 8% of all cancer deaths [1], with variable survival of between 39% and 65% depending on stage at diagnosis [2]. The risk of developing CRC is dependent on both genetic and lifestyle-related factors and increases markedly with age [2]. Although treatment can be curative, a considerable proportion of CRC patients have a high risk of disease recurrence after surgery and chemotherapy [3].

The major pathways implicated in colorectal carcinogenesis include, but are not limited to, the PI3K/mTOR pathway, the mitogen-activated protein kinases (MAPK) pathway, and the Wnt pathway [4], with the JAK/STAT pathway, Hedgehog pathway, and NFκB pathway also involved [5]. These pathways are controlled via complex crosstalk, negative feedback, and other compensatory mechanisms. While activation of these pathways occurs via mutations in participating oncogenes and tumor suppressor genes, respectively, of the 80 somatic mutations in any individual CRC, only 15 or possibly less are likely to be essential drivers of tumor initiation, progression, and/or maintenance [6]. The most frequently mutated genes in CRC are *APC* (70–80%), *TP53* (50%), *KRAS* (35–45%), *PIK3CA* (25–32%), *BRAF* (10–17%) and *PTEN* (4–5%) [7–12].

First line therapy for CRC is usually fluoropyramidine monotherapy and oxaliplatin or irinotecan-based chemotherapy [13]. More recently, monoclonal antibodies such as cetuximab, panitumumab, and bevacizumab have been licensed in combination with chemotherapy for metastatic CRC (mCRC) [14] as selective and specific anticancer agents with a high therapeutic index and lower toxicity than conventional therapies [15]. However, responses to treatment are varied, with less than one-third of patients responding to 5-fluorouracil [16]. Although *KRAS* and *BRAF* mutations indicate resistance to EGFR-targeted therapies, about 40–70% of wild type *KRAS* mCRC patients derive little or no benefit from EGFR-targeted therapies [17]. There remains a lack of predictive markers that allow clinicians to select patients most likely to benefit from a specific therapy.

Here, we sought to systematically characterize a panel of CRC cell lines, selected to reflect the diversity of this disease, using high-throughput analyses in order to identify biomarkers of resistance to both targeted and non-targeted therapies.

## Methods

### CRC cell line panel

Fifteen CRC cell lines were studied: the near diploid cell lines DLD-1, HCT116, HCT116p53-/-, SW48, and LoVo (all from ECACC except HCT116p53-/- which was a gift from Dr G Smith, University of Dundee, UK [18]) and the aneuploid cell lines SW480, SW837, HT29, T84, Colo 201, Colo 320DM, LS411N, SK-CO-1, NCI H508 and NCI H716 (all from ATCC) apart from Colo 320DM, T84, and SW837 (all from ECACC).

The cell lines were cultured in Dulbecco’s modified Eagle’s medium (DMEM) (Gibco^®^, Cat. no. 31885) supplemented with 10% foetal bovine serum (FBS; PAA, Cat. no. A15-101) and 1% penicillin-streptomycin (Gibco^®^, Cat. no.15140-122). The cell lines were grown in a humidified incubator at 37°C containing 5% CO_2_. All the cell lines were tested for mycoplasma using the Venor^™^GeM Mycoplasma Detection Kit (Sigma-Aldrich, Cat. no. MP0025). When the cell lines reached 70–80% confluence, they were trypsinized using 0.05% trypsin-EDTA (1X) with phenol red (Gibco^®^, Cat. no. 25300).

### Clinical samples

Archival formalin-fixed, paraffin-embedded (FFPE) tissue samples were obtained from resection specimens from patients living in Scotland who were diagnosed with CRC between 1996 and 2003 and were under 55 years of age at the time of diagnosis (refer to S1 Table). A total of 870 patients had been recruited as previously described [19]. All cases were reviewed by a gastrointestinal histopathologist prior to TMA construction to ensure that the tissue was comprised primarily of tumor. All cancers were staged Dukes’ A and B. Cohort material and clinical records access was granted by the Tissue Committee, Edinburgh Experimental Cancer Medicine (Ref: TR029), Lothian Research Ethics Committee (Ref: 08/S1101/41) and South East Scotland HSS (SAHSC) BioResource (Ref: SR117).

### Drug sensitivity assays

5-fluorouracil (5-FU) 50mg/mL solution for injection was purchased from Medac GmbH. Oxaliplatin (L-OHP) 5mg/ml concentrate for solution for infusion (Fresenius Kabi Oncology plc, UK) was obtained from the Western General Hospital Pharmacy, Edinburgh. The targeted inhibitor BEZ235 (Cat. no. S1009) was purchased from Selleck Chemicals. Each 96-well plate consisted of six wells containing cells in DMEM supplemented with 10% FBS and 1% penicillin/streptomycin, which served as a control. The cells were seeded for 48h prior to addition of the drugs. Eight different concentrations were used per drug ranging between 5μM to 100μM (5-FU, L-OHP) and between 2.5nM and 80nM, (BEZ235) respectively. The cells were incubated with the drugs for 96h. To determine cell viability, 20μL of Alamar Blue was added in each well for 6h prior to reading the plates using Fluoroskan Ascent FL. All drug sensitivity assays were replicated at least twice and six wells were seeded at each drug concentration.

An average RFU reading was taken for every drug concentration and cell viability was calculated as a percentage of the untreated control. Error bars were calculated using the correlated standard deviation of the means. The IC_50_s for 5-FU, L-OHP and BEZ235 were determined using the XLfit 5.0 software package (ID Business Solutions, UK). No extrapolation was carried out when defining the IC_50_ values and outliers were calculated as having a confidence level greater than 0.05.

### DNA, RNA, and protein extraction

Genomic DNA was extracted from each cell line using DNeasy Blood and Tissue Kit (Qiagen, Cat.No. 69504) according to the manufacturer’s instructions. DNA concentrations were verified using the NanoDrop 2000 micro-volume spectrophotometer (Thermo Scientific). Satisfactory DNA purity was regarded as greater than or equal to a 260/280 ratio of 1.8, ensuring minimal protein contamination of the sample. The quality of the DNA samples was further assayed using agarose gel electrophoresis. After electrophoresis, the gel was carefully removed and the DNA bands were visualised using the Gel Documentation System.

Total RNA was extracted from the cell lines in duplicate using the RNeasy MinElute Cleanup Kit (Qiagen, Cat. no. 74204) and miRNeasy Mini Kit (Qiagen, Cat. no. 217004). The concentration of the RNA was verified using the NanoDrop 2000 spectrophotometer. Satisfactory RNA purity was regarded as a 260/230 ratio of approximately 2.0.

Protein lysates were prepared when the cell lines were approximately 80% confluent, as described in detail elsewhere [20]. The protein concentration of the lysates was determined via the bicinchoninic acid (BCA) assay (Sigma-Aldrich, cat. no. C2284-25ML, cat.no. B9643-1L).

### *KRAS* mutation analysis by Sanger sequencing

Hotspot mutations in codon 12 and 13 were analysed. The primer set was designed using Primer Premier^®^ V6.0 software (PREMIER Biosoft International). The primer sequences (5' to 3') for *KRAS* 01 were as follows: GGT ACT GGT GGA GTA TTT GAT AGT GT (forward) and TGA ATT AGC TGT ATC GTC AAG GCA CT (reverse). *KRAS* exon 2 amplification was carried out using the HotStar Hi Fidelity Polymerase Kit (Qiagen Quality^®^, cat. no. 202602). The PCR reaction was performed in the DNA Engine Opticon 2 Real-Time Cycler (GMI, Inc). The expected length of the PCR product was confirmed by the presence of a single band at the appropriate molecular weight. Sanger sequencing was carried out at the Medical Research Council Human Genetics Unit (MRC-HGU), Edinburgh. Products were sequenced using the ABI Prism^®^ 3100 Genetic Analyzer (Applied Biosystems, Hitachi) and data were analysed using Mutation Surveyor^®^ DNA Variant Analysis V3.97 software.

### Microarray analyses

#### Array comparative genomic hybridization

Comparative genomic hybridization (CGH) was performed using the NimbleGen microarray (Roche). Sample labelling was performed with the NimbleGen Dual-Color DNA Labeling Kit (Roche, cat. no. 06 370 250 001). Hybridization was performed in the MRC-HGU, Edinburgh using a NimbleGen Hybridization Kit (Roche, cat. no. 05 583 683 001), NimbleGen Sample Tracking Control Kit (Roche, cat. no. 05 223 512 001) and two Human CGH 12 x 135K Whole-Genome Tiling Arrays V3.0 (Roche, cat. no. 05 520 878 001). NimbleScan software was used to generate the pair report files used for copy number data analysis. The data have been deposited at the National Centre for Biotechnology Information (NCBI) Gene Expression Omnibus with the accession number GSE72296.

#### Gene expression profiling

Three sets of RNA samples were prepared for Illumina^®^ Whole Genome Gene Expression Profiling, where 48,804 transcripts per sample were generated. The three sets consisted of two sets of biological replicates and one set of technical replicates. All the RNA samples were diluted to a concentration of 500ng/11μl. The Illumina^®^ TotalPrep^™^ RNA Amplification Kit (Ambion^®^, cat. no. AMIL1791) was used to generate biotinylated, amplified RNA for hybridization with the Illumina^®^ Human HT-12 v4.0 BeadChip. Prior to progressing with preparation of the RNA samples for microarray analysis, the RNA integrity was further assessed with the Agilent^®^ 2100 Bioanalyzer using the Agilent^®^ RNA 6000 Nano Kit (Agilent, cat. no. 5067–1511). Samples with an RNA Integrity Number (RIN) of 7 or better were considered acceptable for hybridisation.

The samples were analysed at the Wellcome Trust Clinical Research Facility, Edinburgh (Gene Expression Project—CRF E11960), where they were diluted to a concentration of 150ng/μl and hybridized onto three Human HT-12 v4 Expression BeadChip arrays. Two technical replicates were hybridized onto each array to serve as an internal quality control. The samples were randomly hybridized along the three Illumina^®^ HumanHT-12v4 Expression BeadChip arrays.

Post-hybridization, the arrays were scanned using the Illumina HiScan^®^ Platform (Illumina^®^, cat. no. SY-103-1001). The BeadArray data files were exported from the Illumina’s scanning software and imported into the gene expression module of the GenomeStudio software (Illumina^®^), where subsequently the data files were transformed to tab delimited files. The data have been deposited at the National Centre for Biotechnology Information (NCBI) Gene Expression Omnibus with accession number GSE72544 (http://www.ncbi.nlm.nih.gov/geo/query/acc.cgi?acc=GSE72544).

### Reverse-phase protein arrays

Reverse-phase protein arrays (RPPA) are a medium-throughput technique that allows the screening of samples with a large panel of proteins of interest in a relatively short time, while using minimal amounts of both sample and antibodies [21]. The denatured and reduced protein samples of the 15 CRC samples were spotted in triplicate onto each pad of a 2-Pad FAST^®^ nitrocellulose coated glass slide (Whatman Ltd., cat. no. 10485317) using a BioRobotics MicroGrid MG II Biobank (Isogen Life Science). Subsequently, they were successfully probed with a panel of 31 optimised, in-house validated, total and phospho- antibodies as previously described (S2 Table) [20]. These antibodies were selected to target key proteins involved in cell proliferation and survival, invasion, metastasis, angiogenesis, DNA damage, and apoptosis were optimised and validated via Western Blotting. The RPPA spots were quantified using MicroVigene^™^ RPPA Analysis Module software (VigeneTech Inc.). The data were analysed as previously described [22].

The RPPA spots were quantified using MicroVigene^™^ RPPA Analysis Module software (VigeneTech Inc.).

### Data analysis

#### Genomic data analysis

Sanger sequencing data were analysed using Mutation Surveyor^®^ DNA Variant Analysis Software V3.97 (Soft Genetics^®^, USA). The raw data files .ab1 generated by the ABI Prism^®^ 3100 Genetic Analyzer (Applied Biosystems, Hitachi) were imported into the software and the default analysis settings were applied. The GenBank annotation files were automatically downloaded and the reference files used for mutation detection were automatically synthesised.

aCGH data were analysed using Partek^®^ Genomic Suite^™^ Version 6.6 (Partek Inc.). The data were initially normalised using Loess Normalization and the Genomic Segmentation algorithm was used to analyse the copy number amplifications and deletions. The custom segmentation parameters were as follows: the minimum genomic markers was 10, the p-value was 0.001, and the signal-to-noise ratio was 0.03. A region was reported as lost if the log2 copy number ratio was below -0.3 and gained if the log 2 copy number ratio was above 0.15. Three different region lists were created: (1) regions that were gained in seven or more cell lines; (2) regions deleted in seven or more cell lines; (3) those containing the highest amplifications, i.e., log2 ratio equal or greater to 1.0 (equivalent to a copy number of 2). Additionally, genomic segmentation clustering was performed using Euclidean distance and average linkage. The copy number analysis was conducted on chromosome 1 to chromosome 22 and excluded the two sex chromosomes.

#### Transcriptomic data analysis

The sample gene profile file generated from the gene expression analysis was quantile normalised and filtered for those probes where the detection p-value ≤0.05. The data were then log2 transformed and mean centred to obtain relative values between the cell lines. The sample gene profile file was then annotated using Hg18 prior to performing differential gene expression analysis (DGEA).

The DGEA was performed using ArrayMining, an online microarray data mining software package [23]. Differential gene expression was conducted using SAM analysis to list genes differentially expressed with respect to treatment response. Three different analyses were carried out: (1) 5-FU highly sensitive cell lines *vs*. 5-FU less sensitive cell lines, where highly sensitive cell lines were defined as having an IC_50_ ≤ 30μM; (2) L-OHP highly sensitive cell lines *vs*. L-OHP less sensitive cell lines, where highly sensitive cell lines were defined as having an IC_50_ ≤ 10μM; (3) BEZ235 sensitive cell lines *vs*. BEZ235 insensitive cell lines, where sensitive cell lines were defined as having an IC_50_ < 80nM.

Interpretation of data was accomplished using Functional Annotation Clustering in DAVID bioinformatics resources [24].

#### Integration of frequently amplified regions with gene expression data

The gene expression data for the genes located in the frequently gained regions was filtered out. Using Pearson’s correlation coefficients with Bonferroni correction, a list of genes that had a significant correlation between the log2 copy number value and gene expression was generated. The gene expression data for cell lines were analysed with respect to treatment response using Mann-Whitney U test using GraphPad Prism 6.

#### Proteomic data analysis

Data generated from RPPA were normalised using Cluster 3.0, an open source clustering tool [25]. Data were log-transformed, mean centred in Cluster 3.0, and clustered by correlation centring and average linkage using MeV 4.8 [26]. RPPA results for the 15 CRC panel were analysed with respect to treatment response using Mann Whitney U test using GraphPad Prism 6.

### Tissue microarray (TMA), automated quantitative analysis (AQUA), and FISH

Five-micron haematoxylin and eosin-stained slides were prepared from the FFPE blocks, and tumor areas were marked by a pathologist and a trained research technician. Following histopathological examination, 118 cases were chosen out of the original cohort and a tissue microarray (TMA) was constructed by a qualified technician. Four biological replicates (TMA000034A-D) were constructed as described in detail elsewhere [27] and cut into 5μm sections using a microtome and mounted onto glass slides. Clinical and pathological parameters of this cohort are summarised in S1 Table.

Protein expression of TRIB1 was assessed with anti-TRIB1 rabbit polyclonal antibody in the CRC TMA using Automated QUantitative Analysis (AQUA), described in detail elsewhere [28,29]. TRIB1 expression in both the cytoplasmic and nuclear compartments was subsequently correlated with other proteins previously measured in this cohort. TRIB1 expression was also investigated with respect to patient survival, as described below.

*TRIB1* and *MYC* amplification in the CRC patient cohort were investigated using fluorescence *in situ* hybridisation (FISH). A MYC/CEN8p probe was purchased from Abnova (cat. no. FG0065) and the TRIB1/CEN8p probe was custom designed by Abnova. The protease treatment time was varied to optimise digestion and ensure good quality hybridisation. Visualisation was performed using DAPI (4,6-diamidino-2-phenylindole-2-hydrochloride (Abnova) to stain nuclei.

Ready-to-use dual-labelled probes for *MYC* and *TRIB1* were purchased from Abnova. The MYC/CEN8p FISH probe consisted of an ~160kb *MYC* probe located at 8q24.12-q24.13 with a Texas Red fluorophore together with an ~520kb CEN8p probe located at 8p11.21 with a FITC fluorophore. The TRIB1/CEN8p FISH probe consisted of an ~260kb *TRIB1* probe located at 8q24.13 with a Texas Red fluorophore together with an ~520kb CEN8p probe located at 8p11.21 with a FITC fluorophore.

Scoring was carried out by a trained technician and a consultant pathologist. The slides were scored using a Leica DMLB fluorescent microscope using 100X oil immersion lens. The Colorado Scoring Criteria were used [30] to score the TMA slides. A maximum of twenty nuclei per core were scored in most cases, although in some cases a minimum of ten nuclei were scored due to not having twenty scorable nuclei. The sum of the red and green fluorophores was noted for each core, and the final score consisted of the ratio of the red fluorophore to the green fluorophore. FISH scores less than 1.8 were interpreted as negative [31].

### Statistical analyses

TRIB1 protein expression data generated from AQUA analysis were correlated with AKT, caspase 3, cyclin B1, ERK, Ki67, MYC, S6, PTEN, pAKT, pERK, pHistone H3, pMEK, and pS6 protein expression. Statistical analysis was carried out using Pearson’s correlations, and p-values were adjusted for multiple testing using the Bonferroni correction. An open source programme TMA Navigator (http://www.tmanavigator.org/) was used for statistical analysis. Survival analysis for *TRIB1* and *MYC* amplification in the CRC cohort was carried out using GraphPad Prism 6.

## Results

### Single gene mutational analysis is insufficient for stratification of tumors with respect to therapy

**Fig 1:**
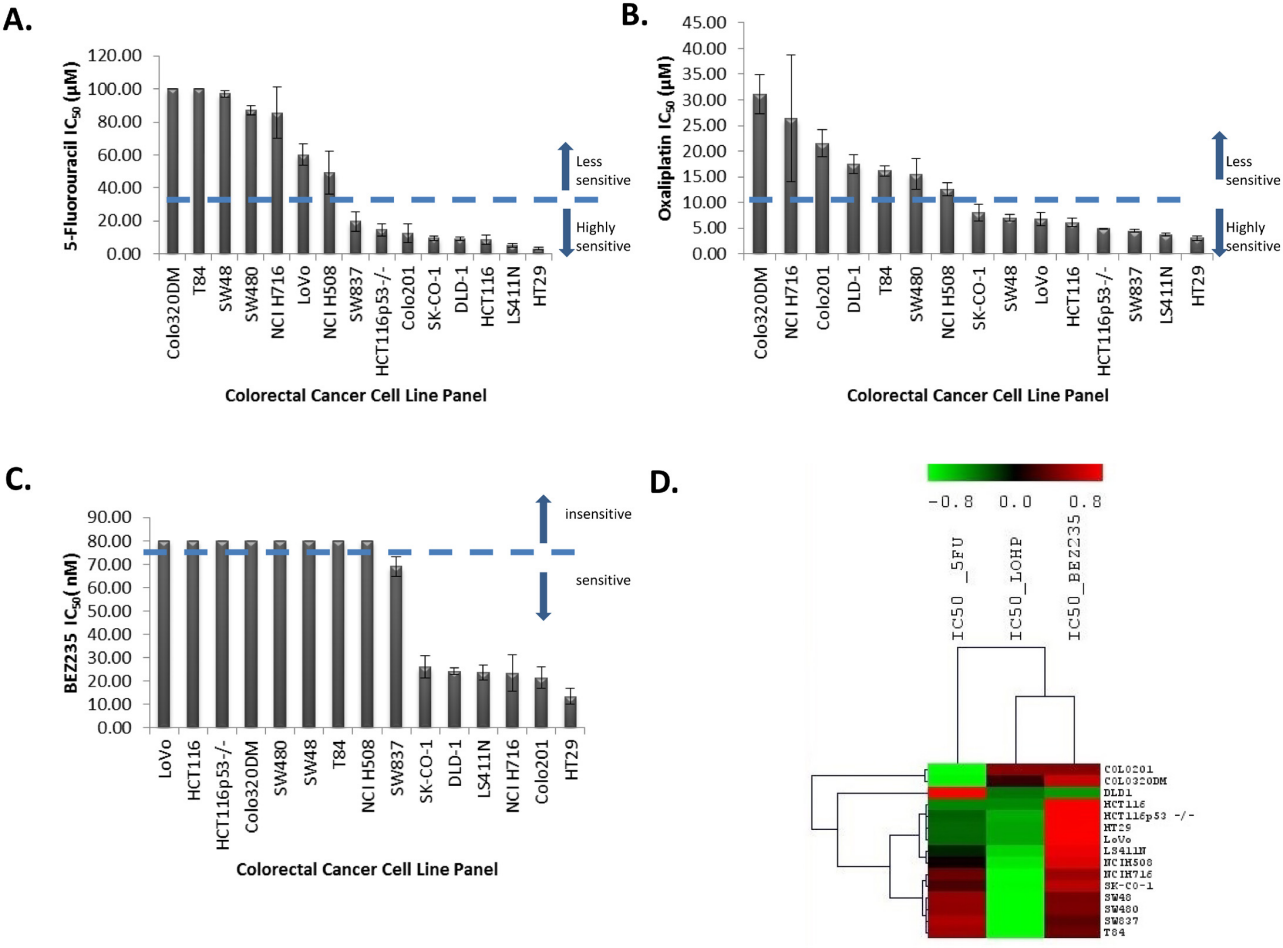
A. Waterfall plot for the 5-fluorouracil IC_50_ (μM) values. B. Waterfall plot for oxaliplatin IC_50_ (μM) values. C. Waterfall plot for BEZ235 IC_50_ (nM) values. D. Unsupervised hierarchical clustering for the IC_50_ values for 5-FU, L-OHP and BEZ235 using Pearson’s Correlation with complete linkage.

After treatment with 5-fluorouracil (5-FU) for 96 h, thirteen CRC cell lines showed varying degrees of sensitivity when treated with drug concentrations ranging from 2.5μM to 100μM (Fig 1A). Two CRC cell lines (Colo320DM, T84) were insensitive to 5-FU at a concentration of 100μM. The IC_50_ values for 5-FU ranged from 3.1 to >100μM with a median of 19.6μM. The most sensitive cell lines were HT29, LS411N, and HCT116. DLD-1, HCT116, HCT116p53-/-, SW48, and LoVo are reported to be mismatch repair deficient [32]. This profile of mismatch repair status did not correlate with 5-FU sensitivity (p = 0.713; Mann-Whitney U test) contrary to a study by Bracht and colleagues [33].

Although a number of *in vitro* studies have suggested that *TP53* deficiency contributes to drug resistance [34], we failed to see an association (p = 0.238; Mann-Whitney U test). HT29, LS411N, HCT116 p53-/-, SW837, NCI H508, NCI H716 are all *TP53* deficient (http://cancer.sanger.ac.uk/cancergenome/projects/cosmic/), but they were still sensitive to 5-FU in this study. Mariadason et al., however, reported no difference in 5-FU-induced apoptosis in mutant and wild type p53 cell lines [35].

After treatment with oxaliplatin (L-OHP) for 96 h, the CRC cell lines showed varying degrees of sensitivity when treated with increasing concentrations of L-OHP ranging from 2.5μM to 100μM (Fig 1B). The IC_50_ values for L-OHP ranged from 3.0 to 31.1μM, with a median of 8.0 μM, demonstrating a ten-fold range of sensitivity. The most sensitive cell lines were HT29, LS411N, and SW837, while the least sensitive were Colo320DM, NCI H716, and Colo201. No statistical significance (p = 0.462; Mann Whitney U Test) was observed when comparing L-OHP IC_50_ values between dMMR cell lines and pMMR cell lines, which is in agreement with a similar study by Fink et al. [36].

There was no association between p53 status and L-OHP IC_50_ values (p = 0.187; Mann Whitney U Test), in contrast to a previous report [37]. However, a recent study carried out in 51 advanced CRC patients concluded that *TP53* mutational status was not associated with benefit from first-line oxaliplatin-based treatment [38].

Seven CRC cell lines were sensitive and eight CRC cell lines were insensitive to treatment with various concentrations (2.5nM and 80nM) of BEZ235 for 96 h (Fig 1C). The IC_50_ values for BEZ235 ranged from 13.4 to >80nM, with the sensitive cell lines having a median sensitive concentration of 23.6nM. The most sensitive cell lines were HT29, Colo201, and NCI H716, while NCI H508, T84, SW48, SW480, Colo320DM, HCT116, HCT116 p53-/-, and LoVo were insensitive at a concentration of 80nM. No statistically significant difference was observed (p = 0.346; the Mann-Whitney U test) between the IC_50_ values for BEZ235 treatment and *PIK3CA* mutant and wild type groups. All the *PI3KCA* mutant cell lines had either a *BRAF* or a *KRAS* co-mutation. No COSMIC data was available for *MTOR* mutations in these cell lines (http://cancer.sanger.ac.uk/cancergenome/projects/cosmic/). Serra et al. established that BEZ235 arrested proliferation in all 21 cancer cell lines used in their study, independent of PI3K pathway mutation status [39], and that cell lines with a *BRAF* or *KRAS* mutation or *EGFR* amplification were slightly less sensitive to BEZ235 compared to the other cell lines [39].

Of the 15 CRC cell lines, eight cell lines possessed *KRAS* exon 2 mutations. The DLD-1, HCT116, HCT116 p53-/-, and LoVo cell lines had a 5574 G>A substitution consistent with a G13D missense mutation; the SK-CO-1 and SW480 cell lines had a 5571 G>T substitution consistent with a G12V missense mutation; SW837 had a 5570 G>T substitution consistent with a G12C mutation; and T84 had a 5574 G>A substitution consistent with a G13D mutation. This is in agreement with published sequencing data and data in the COSMIC database (http://cancer.sanger.ac.uk/cancergenome/projects/cosmic/). There were no statistically significant differences in response to 5-FU, L-OHP, and BEZ235 with respect to *KRAS* mutational status (p = 0.98, p = 0.60, and p = 0.17, respectively).

Unsupervised hierarchical clustering for the IC_50_ values for 5-FU, L-OHP, and BEZ235 using Pearson’s correlations with complete linkage showed that the cell lines did not cluster according to any particular mutation. There was variability in response to the three different treatments (Fig 1D).

### Chromosomal regions frequently gained and lost in the colorectal cancer cell lines

**Fig 2:**
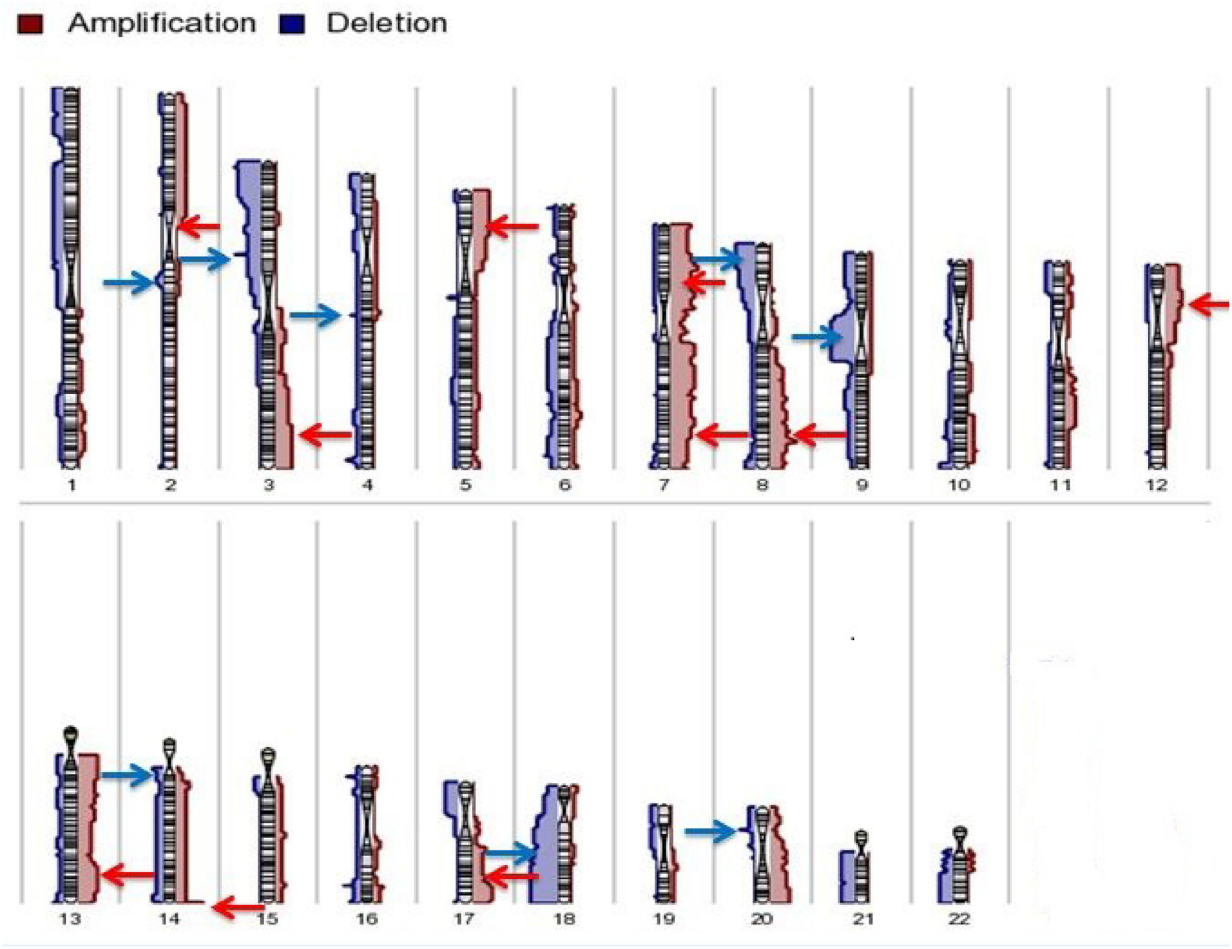
Karyogram for chromosome 1 to 22 showing the most frequent gains and losses for the 15 CRC cell lines

The panel of cell lines was next evaluated by aCGH to identify common chromosomal regions of gain and loss. Twenty-four regions were frequently gained in at least 7/15 CRC cell lines. Frequent gains were observed at 2p, 3q, 5p, 7p, 7q, 8q, 12p, 13q, 14q, and 17q (Fig 2) (Table 1). On the other hand, a total of 14 regions were lost in at least 7/15 CRC cell lines (Table 2). Frequent losses were observed at 2q, 3p, 5q, 8p, 9p, 14q, 18q, and 20p (Fig 2). These regions of gain and loss were similar to those previously reported [32, 40–44].

**Table 1:** Summary of the regions of copy number gains and the genes significantly overexpressed in those regions (* after Bonferroni correction).

**Cell lines containing amplicons**	**Cytoband**	**Starting bp**	**Ending bp**	**Size (Mb)**	**aCGH copy number gains range (log**_**2**_ **ratio)**	Significantly correlated over-expressed genes found in the amplicon*
Colo201, Colo320DM, HCT116, HCT116 p53-/-, HT29, LS411N, LoVo, NCI H508, NCI H716, SK-CO-1, SW480, SW837, T84	2p11.2	88,741,497	89,240,742	0.5	0.2–1.1	
Colo320DM, HT29, LS411N, NCI H508, NCI H716, SW480, T84	3q26.32	180,172,618	180,364,415	0.02	0.2–0.6	
Colo201, Colo320DM, HT29, LS411N, NCI H508, SW480, T84	3q27.1	185,357,499	185,500,005	0.14	0.2–1.2	
Colo320DM, HCT116, HCT116 p53-/-, HT29, LS411N, NCI H508, SW480, T84	3q28	190,361,018	190,756,766	0.4	0.2–0.7	
Colo201, Colo320DM, HT29, LS411N, NCI H508, SW480, T84	3q29	198,737,465	198,973,765	0.24	0.2–0.7	
Colo201, HT29, LS411N, LoVo, NCI H508, NCI H716, SK-CO-1, SW480	5p15.33-p14.1	144,656	25,059,988	24.92	0.2–0.7	*PDCD6*
Colo201, LoVo, NCI H508, NCI H716, SW48, SW480, T84	7p22.3	959,839	1,554,223	0.59	0.3–0.8	
DLD-1, LoVo, NCI H508, NCI H716, SW48, SW480, T84	7p21.3	10,059,941	10,462,006	0.40	0.2–1.1	
Colo201, LS411N, LoVo, NCI H508, NCI H716, SW48, SW480, T84	7p21.1-p14.2	19,159,609	36,992,541	13.35	0.3–0.9	*CYCS*, *TOMM7*, *MIR196B*, *NOD1*
Colo201, LS411N, LoVo, NCI H508, NCI H716, SK-CO-1, SW48, SW480, T84	7p14.2-p11.2	37,182,752	56,225,015	19.04	0.2–0.6	*MRPL32*, *DDX56*,*PURB*, *TBRG4*,*COBL*, *LANCL2*,*MRPS17*
LS411N, LoVo, NCI H508, SK-CO-1, SW48, SW480, T84	7q11.22	69,497,636	71,538,673	2.04	0.2–0.3	
Colo201, DLD-1, HT29, LS411N, LoVo, NCI H508, NCI H716, SK-CO-1, SW48, SW480, T84	7q11.23–31.1	76,596,876	110,404,588	33.81	0.2–1.1	*TMEM60*, *CLDN12*, *SHFM1*, *LMTK2*, *PTCD1*,*PLOD3*, *ZNHIT1*, *ARMC10*, *RINT1*, *BCAP29*, *SLC26A4*,
**Cell lines containing amplicons**	**Cytoband**	**Starting bp**	**Ending bp**	**Size (Mb)**	**aCGH copy number gains range (log**_**2**_ **ratio)**	Significant correlated over-expressed genes found in the amplicons*
LS411N, LoVo, NCI H508, NCI H716, SK-CO-1, SW48, T84	7q31.1-q31.31	110,930,968	119,328,807	8.4	0.2–0.6	*ST7*
Colo201, HCT116 p53-/-, LS411N, LoVo, NCI H508, NCI H716, SK-CO-1, SW48	7q31.33-q34	124,724,896	138,981,311	14.26	0.2–1.0	*IMPDH1*, *CHCHD3*, *NUP205*, *KIAA1549*, *LUC7L2*
Colo201,LoVo, NCI H508, NCI H716, SK-CO-1, SW48, T84	7q35	146,954,947	147,418,309	0.46	0.2–0.6	
Colo201, Colo320DM, HCT116, HCT116 p53-/-, HT29, NCI H716, SK-CO-1, SW480, SW837	8q24.13-q24.21	126,328,971	128,964,088	1.46	0.3–4.2	*NSMCE2*, *TRIB1*, *FAM84B*, *LOC727677*, *MIR1204*, *MYC*
Colo201, Colo320DM, HCT116, HCT116 p53-/-, HT29, SK-CO-1, SW480	8q24.21	129,068,127	129,110,839	0.04	0.3–3.4	
Colo201, LS411N, LoVo, NCI H716, SW480, SW837, T84	12p13.3	33,393	185,534	0.15	0.2–0.6	
Colo320DM, LS411N, LoVo, NCI H716, SK-CO-1, SW480, SW837, T84	12p12.3-p12.2	15,652,223	20,311,064	4.66	0.2–1.5	*STRAP*, *AEBP2*
Colo201, Colo320DM, HT29, LS411N, NCI H508, NCI H716, SK-CO-1, SW480	13q12.11-q13.3	18,761,622	35,141,488	16.38	0.2–3.39	*MPHOSP8*, *N6AMT2*, *XPO4*, *GTF3A*, *MTIF3*, *POMP*, *UBL3*, *BRCA2*, *PDS5B*, *RFC3*
Colo201, Colo320DM, DLD-1, HT29, LS411N, NCI H508, SW480	13q14.11	42,472,749	42,745,298	0.27	0.2–0.8	
Colo201, Colo320DM, HCT116, HCT116 p53-/-, HT29, LS411N, LoVo, NCI H508, NCI H716, SK-CO-1, SW48, SW480, SW837, T84	14q32.33	105,305,751	106,342,077	1.04	0.4–1.3	
Colo201, Colo320DM, HCT116, HCT116 p53-/-, HT29, NCI H508, SW837	17q24.1	61,037,879	61,181,176	0.14	0.2–0.7	
Colo201, HCT116, HCT116 p53-/-, NCI H508, NCI H716, SK-CO-1, SW837	17q25.1	70,481,449	70,707,547	0.23	0.3–1.0	

**Table 2:** Summary of the regions having copy number losses.

Cell lines containing deletions	Cytoband	Starting bp	Ending bp	Size (Mb)	aCGH copy number deletions range (log_2_ ratio)	Candidate genes in the regions of copy number loss
Colo201, Colo320DM, LS411N, NCI H508, SK-CO-1, SW480, T84	2q23.3	152128419	152319262	0.2	-0.3 to -0.65	*NEB*
Colo201, HCT116 p53-/-, HT29, LS411N, NCI H508, NCI H716, SW837	3p14.2	60179044	60195847	0.02	-0.3 to -1.6	Intron of FHIT
Colo201, HCT116, HCT116 p53-/-, HT29, LS411N, NCI H508, NCI H716, SW837	3p14.2	60195847	60211085	0.02	-0.3 to -1.6	Intron of FHIT
Colo201, HCT116, HCT116 p53-/-, HT29, LS411N, LoVo, NCI H508, NCI H716, SW837	3p14.2	60211085	60366651	0.2	-0.3 to -3.0	Intron of FHIT
Colo201, HCT116 p53-/-, HT29, LS411N, LoVo, NCI H508, NCI H716, SW480, SW837	3p14.2	60366651	60600423	0.2	-0.3 to -3.0	*FHIT*
Colo201, HCT116 p53-/-, HT29, LS411N, LoVo, NCI H508, NCI H716, SW837	3p14.2	60600423	60601597	0.001	-0.3 to -2.2	Intron of FHIT
Colo201, Colo320DM, HCT116 p53-/-, HT29, LS411N, LoVo, NCI H508, NCI H716, SW837	3p14.2	60601597	60659727	0.06	-0.3 to -2.2	Intron of FHIT
Colo201, Colo320DM, HCT116 p53-/-, HT29, LS411N, NCI H716, SW837	3p14.2	60659727	60699679	0.04	-0.3 to -2.2	Intron of FHIT
Colo201, LS411N, LoVo, SK-CO-1, SW480, SW837, T84	5q13.2	68918436	69002998	0.08	-0.4 to -0.7	SMA4, GTF2H2B, GTF2H2C, GTF2H2D, GTF2H2, GUSBP3, LOC100272216
Colo201, Colo320DM, HT29, LS411N, LoVo, SK-CO-1, SW480, SW837, T84	5q13.2	69002998	69127115	0.1	-0.4 to -0.7	contained within SMA4, region overlaps with 34.27% of GUSBP3
Colo201, Colo320DM, HT29, LS411N, LoVo, SK-CO-1, SW48, SW480, SW837, T84	5q13.2	69127115	69684303	0.6	-0.4 to -0.7	
Colo201, Colo320DM, HT29, LS411N, LoVo, SK-CO-1, SW480, SW837, T84	5q13.2	69684303	70543264	0.9	-0.4 to -0.7	SMA4, GTF2H2B, GTF2H2C, GTF2H2D, SMA5, LOC441081, GUSBP9, SERF1A, SERF1B, SMN2, SMN1, NAIP, LOC647859
Colo201, Colo320DM, HT29, LS411N, LoVo, SK-CO-1, SW480, SW837	5q13.2	70543264	70669127	0.1	-0.4 to -0.7	GUSBP9
HT29, NCI H508, NCI H716, SK-CO-1, SW480, SW837, T84	8p23.1	6759882	6779798	0.02	-0.3 to -1.4	DEFA6, region ends 957 bp before DEFA4
Colo320DM, HT29, LS411N, NCI H508, NCI H716, SK-CO-1, SW480, SW837, T84	8p23.1	6779798	6824457	0.04	-0.5 to -1.4	DEFA10P, DEFA4, region overlaps with 4.20% of DEFA1, region overlaps with DEFA1B
Colo201, Colo320DM, HT29, LS411N, NCI H508, NCI H716, SK-CO-1, SW480, SW837, T84	8p23.1	6824457	7196061	0.4	-0.5 to -1.4	
Colo201, Colo320DM, HT29, LS411N, NCI H716, SK-CO-1, SW480, SW837, T84	8p23.1	7196061	7243352	0.05	-0.5 to -1.2	ZNF705G, region overlaps with 8.94% of FAM66B
Colo201, Colo320DM, HT29, LS411N, NCI H716, SK-CO-1, SW48, SW480, SW837, T84	8p23.1	7243352	7760349	0.5	-0.5 to -1.2	
Colo201, Colo320DM, HT29, LS411N, NCI H716, SK-CO-1, SW480, SW837, T84	8p23.1	7760349	7767962	0.01	-0.5 to -1.2	region ends 8174 bp before DEFB103A
Colo201, Colo320DM, HT29, NCI H716, SK-CO-1, SW480, SW837, T84	8p23.1	7767962	8024923	0.3	-0.4 to -1.2	DEFB103A, DEFB103B, DEFB109P1B, DEFB4A, FAM66E, MIR548I3, USP17L3, USP17L8, ZNF705B
HT29, LS411N, NCI H716, SK-CO-1, SW480, SW837, T84	8p23.1	11368117	11512387	0.1	-0.3 to -1.0	BLK, LINC00208
HT29, NCI H508, NCI H716, SK-CO-1, SW480, SW837, T84	8p22	15174627	15414385	0.2	-0.3 to -0.8	region ends 27582 bp before TUSC3
HCT116, HT29, LoVo, NCI H508, NCI H716, SW48, T84	9p12	41613166	41759552	0.1	-0.4 to -1.1	region starts 30958 bp after ZNF658B
Colo320DM, HCT116, HT29, LoVo, NCI H508, NCI H716, SW48, T84	9p12-11.2	41759552	43003659	1.2	-0.4 to -1.1	MGC21881, KGFLP2, LOC643648, ANKRD20A2, ANKRD20A3, FAM95B1, FOXD4L4, FOXD4L2, LOC286297, AQP7P3
Colo320DM, HT29, LoVo, NCI H508, NCI H716, SW48, T84	9p11.2	43003659	43678360	0.7	-0.4 to -1.1	ANKRD20A2, ANKRD20A3, FAM95B1, LOC642929, FAM75A6, CNTNAP3B
Colo320DM, HT29, LoVo, NCI H508, NCI H716, SW48, T84	9p11.2 –q13	43794421	70017489	26.2	-0.4 to -1.1	CNTNAP3B, LOC643648, FAM27C, FAM27A, KGFLP1, FAM74A4, FAM74A2, SPATA31A5, SPATA31A7, MGC21881, LOC28627, AQP7P1, FAM27B, ANKRD20A1, ANKRD20A3, LOC642236, LOC100132352, PGM5P2, LOC440896, FOXD4L6, CBWD6, ANKRD20A4, LOC100133920, FOXD4L5, FOXD4L2, FOXD4L4, CBWD3, CBWD5
HT29, LS411N, LoVo, NCI H508, NCI H716, SW48, SW480, SW837, T84	14q11.1-q11.2	18407780	19456314	1.0	-0.3 to -1.4	LOC642426, OR11H12, OR11H2,OR4K2, OR4M1, OR4N2, OR4Q3, POTEG, POTEM
Colo201, DLD-1, LS411N, NCI H716, SW480, SW837, T84	18q21.1	43485291	44789986	1.3	-0.3 to -0.9	CTIF, MIR4743, SMAD2, SMAD7, ZBTB7C
Colo201, LS411N, NCI H508, NCI H716, SW480, SW837, T84	18q21.1	45939166	46237377	0.3	-0.4 to -0.9	CCDC11, CXXC1, MBD1, SKA1, region overlaps with 12.17% of MYO5B
Colo201, LS411N, NCI H716, SK-CO-1, SW480, SW837, T84	18q21.2	47301645	49512571	2.2	-0.4 to -0.9	DCC, region overlaps with 1.86% of LOC100287225
Colo201, LS411N, NCI H716, SK-CO-1, SW480, SW837, T84	18q21.2-q23	51105332	76108541	25.0	-0.4 to -1.1	TCF4, MIR4529, LOC100505474, TXNL1, WDR7, LINC-ROR, BOD1L2, ST8SIA3, ONECUT2, FECH, NARS, LOC100505549, ATP8B1, NEDD4L, MIR122, MIR3591, ALPK2, MALT1, ZNF532, OACYLP, SEC11C, GRP, RAX, CPLX4, LMAN1, CCBE1, PMAIP1, MC4R, CDH20, RNF152, PIGN, KIAA1468, TNFRSF11A, ZCCHC2, PHLPP2, BCL2, KDSR, VPS4B, SERPINB5, SERPINB12, SERPINB13, SERPINB4, SERPINB3, SERPINB11, SERPINB7, SERPINB2, SERPINB10, HMSD, SERPINB8, LINC00305, LOC284294, LOC400654, CDH7, CDH19, MIR5011, DSEL, LOC643542, TMX3, CCDC102B, DOK6, CD226, RTTN, SOCS6, LOC100505776, CBLN2, NETO1, LOC400655, LOC100505817, FBX015, TIMM21, CYB5A, C18ORF63, FAM69C, CNDP2, CNDP1, LOC400657, ZNF407, ZADH2, TSHZ1, C18ORF62, LOC339298, ZNF516, FLJ44313, LOC284276, LOC100131655, ZNF236, MBP, GALR1, SALL3, ATP9B, NFATC1, CTDP1, KCNG2, PQLC1, HSBP1L1, TXNL4A, RBFA, ADNP2, PARD6G-AS1, PARD6G
Colo201, HT29, NCI H508, NCI H716, SW480, SW837, T84	20p12.1	14636068	14938351	0.3	-0.4 to -3.2	MACROD2

**Fig 3:**
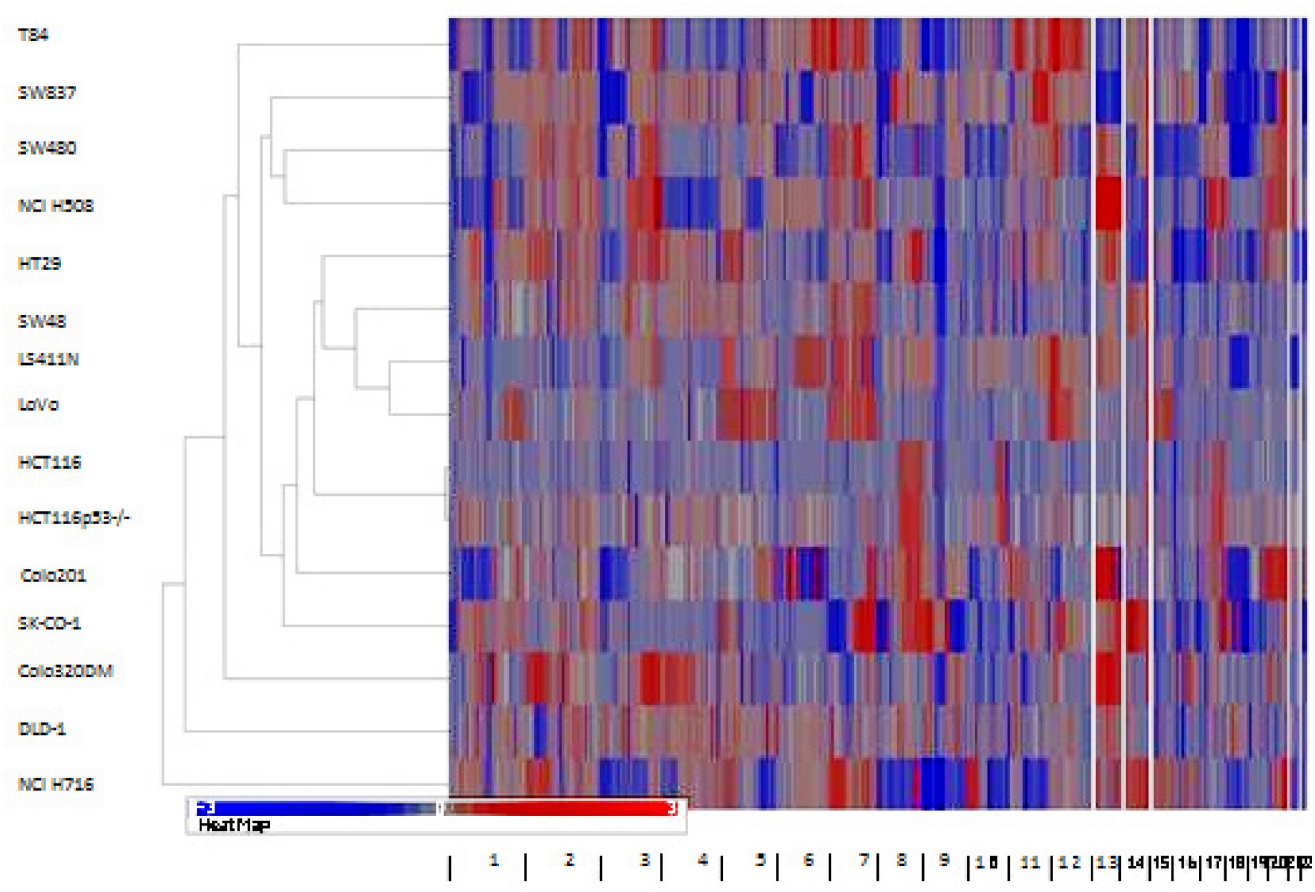
Hierarchical clustering using the genomic segmentations of the 15 CRC cell lines.

Hierarchical clustering of the segmented copy number data using Euclidean distance average linkage resulted in two major clusters: one cluster contained NCI H716 while the other cluster contained the other 14 cell lines (Fig 3). One of the sub-clusters contained HT29, SW48, LS411N, LoVo, HCT116, and HCT116p53-/-. HCT116, HCT116p53-/-, SW48, and LoVo are near-diploid and known to have mutations in MMR genes *MLH1* and *MSH2* [45, 46]. The other near-diploid cell line, DLD-1, also clustered separately. This cell line is MMR deficient in MSH6 [47].

### Differential gene expression with respect to drug sensitivity

**Fig 4:**
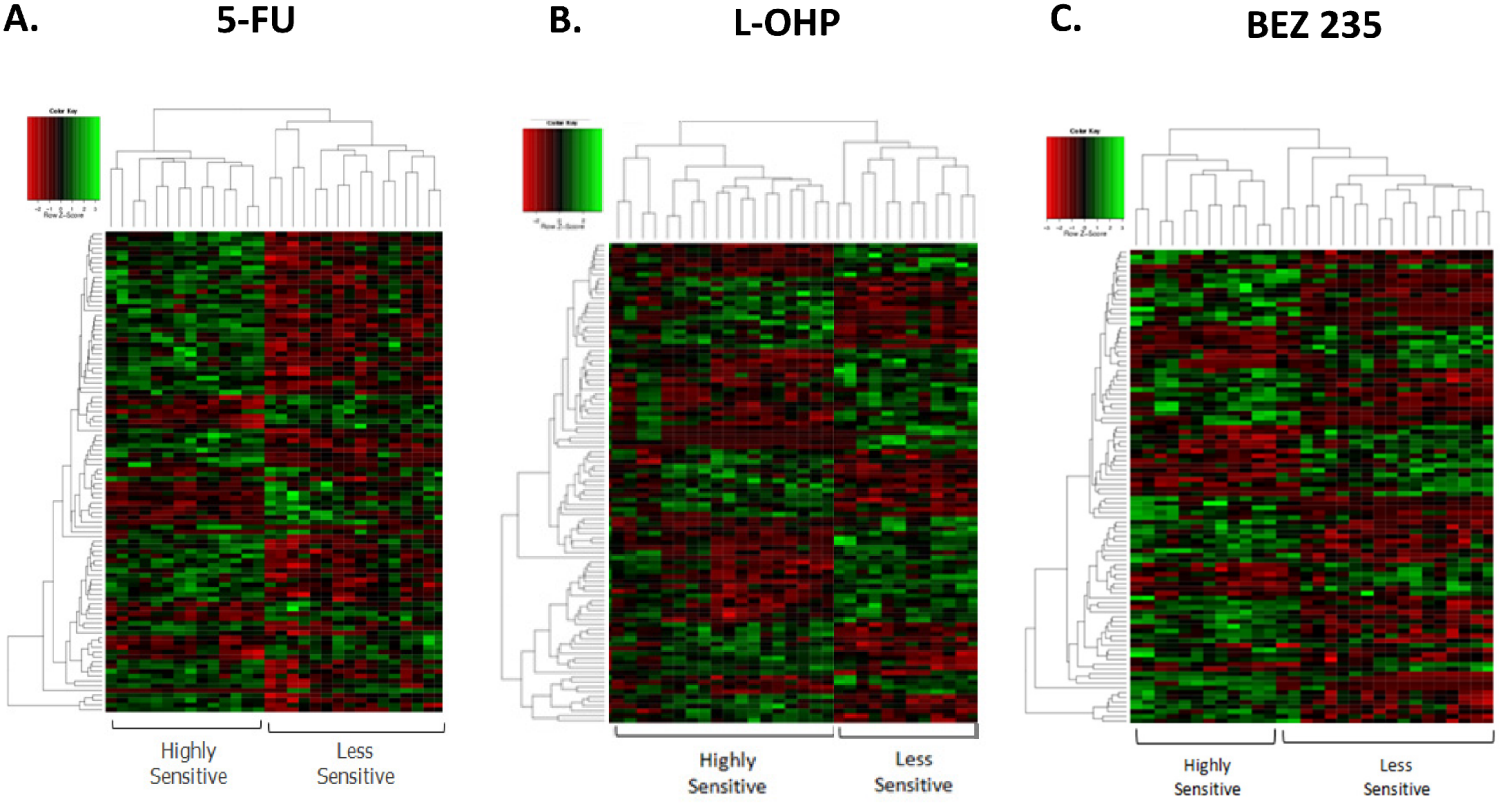
A. A heatmap depicting the SAM analysis for genes differentially expressed between 5-FU sensitive and less sensitive CRC cell lines; B. A heatmap depicting the SAM analysis for genes differentially expressed between L-OHP sensitive and less sensitive CRC cell lines; C. A heatmap depicting the SAM analysis for genes differentially expressed between BEZ235 sensitive and less sensitive CRC cell lines.

Genes differentially expressed with respect to 5-FU sensitivity are listed in S3 Table and depicted in a heat map in Fig 4A. Functional annotation using DAVID [24] revealed that these genes were mainly involved in cell cycle (*TAF2*, *CHFR*, *CCND2*, *OSGIN2*, *TERF1*, *TBRG4*), focal adhesion (*ABCB1*, *SH3KBP1*, *EBAG9*), apoptosis (*SHRKBP1*, *EBAG9*, *TERF1*, *TBRG4*), and regulation of transcription (*LASS2*, *LMCD1*, *MAF1*, *TAF2*, *THAP11*, *CHURC1*, *MED14*, *PIAS3*, *PURB*, *TERF1*, *ZNF239*, *ZNF7*). Important KEGG pathways associated with 5-FU mode of action and subsequently enriched in the list originating from this study included purine metabolism (*NT5C2*, *POLR2J2*), pyrimidine metabolism (*NT5C2*, *POLR2J2*), drug metabolism (*GSTO2*), ABC transporters (*ABCB1*), and oxidative phosphorylation (*NDUFA9*).

Genes differentially expressed with respect to L-OHP sensitivity were involved with DNA binding (*GLI2*, *GLI4*, *SETDB2*, *NFXL1*, *POLE4*, *PURA*, *TSNAX*, *ZBTB41*, *ZNF20*, *ZNF254*, *ZNF420*, *ZNF689*, *ZNF7*, *ZNF91*), regulation of transcription (*GLI2*, *NFXL1*, *PURA*, *TGFBRAP1*, *ZBTB41*, *ZNF20*, *ZNF254*, *ZNF420*, *ZNF689*, *ZNF7*, *ZNF91*), regulation of cell cycle (*CHFR*, *RPS27L*, *SCRIB*, *TPR*), and apoptosis (*BFAR*, *EIF2AK2*, *SCRIB*, *TNFSF9*) (S4 Table and Fig 4B). Oxidative phosphorylation (*ATP6V1B2*), Jak-STAT signalling pathway (*CBLC*), hedgehog signalling pathway (*GLI2*), glycolysis (*AKR1A1*), glutathione metabolism (*GSTO2*), drug metabolism (*GSTO2*), cysteine and methionine metabolism (*MTAP*), MAPK signalling pathway (*MAP3K2*, *MAP4K2*), base excision repair, and nucleotide excision repair (*POLE4*) pathways were enriched in this gene set.

The most differentially expressed genes with respect to BEZ235 sensitivity were involved in glucose metabolism (*CPS1*, *G6PD*, *PYGL*), cell death (*TRIAP1*, *ERN2*, *LYZ*, *MUC5AC*, *PPT1*, *PTRH2*, *RNF216*), response to drug (*TIMP4*, *AACS*, *CPS1*), chromatin organization (*BCORL1*, *LOC644914*, *LOC440926*, *H3F3A*, *SMARCC1*, *TBL1XR1*), regulation of transcription (*BCORL1*, *LMCD1*, *SPDEF*, *SMARCC1*, *TAF4B*, *CHURC1*, *ERN2*, *PROX1*, *SORBS3*, *TBL1XR1*, *ZNF75A*), and DNA binding (*LOC644914*, *LOC440926*, *H3F3A*, *SPDEF*, *SMARCC1*, *TAF4B*, *MSRB2*, *NUCB1*, *PROX1*, *TBL1XR1*, *ZNF75A*) (Fig 4C and S5 Table). The Wnt signalling pathway (*LRP5*, *TBL1XR1*), phosphatidylinositol signalling system (*PIK3C2B*), and the Jak-STAT signalling pathway (*SPRY1*) were enriched in less sensitive cell lines.

### Integration of frequently amplified regions with gene expression data

A total of 971 genes were located in frequently gained regions, of which corresponding gene expression data were available for 667 genes. A total of 47 genes were significantly correlated and are listed in Table 1, suggesting that at least 7% of the genes found in the frequently gained regions might be regulated by copy number changes, at least in part. This is important since genes that are over-expressed when amplified are more likely to be putative oncogenic drivers and therapeutic targets [48]. These amplified and overexpressed genes were involved in pathways in cancer, colorectal cancer drug metabolism, cell cycle, homologous recombination, DNA replication, nucleotide excision repair, mismatch repair, apoptosis, p53 signalling, MAPK signalling, ErbB signalling, wnt signalling, TGF-beta signalling, and JAK-STAT signalling by pathway analysis.

**Fig 5:**
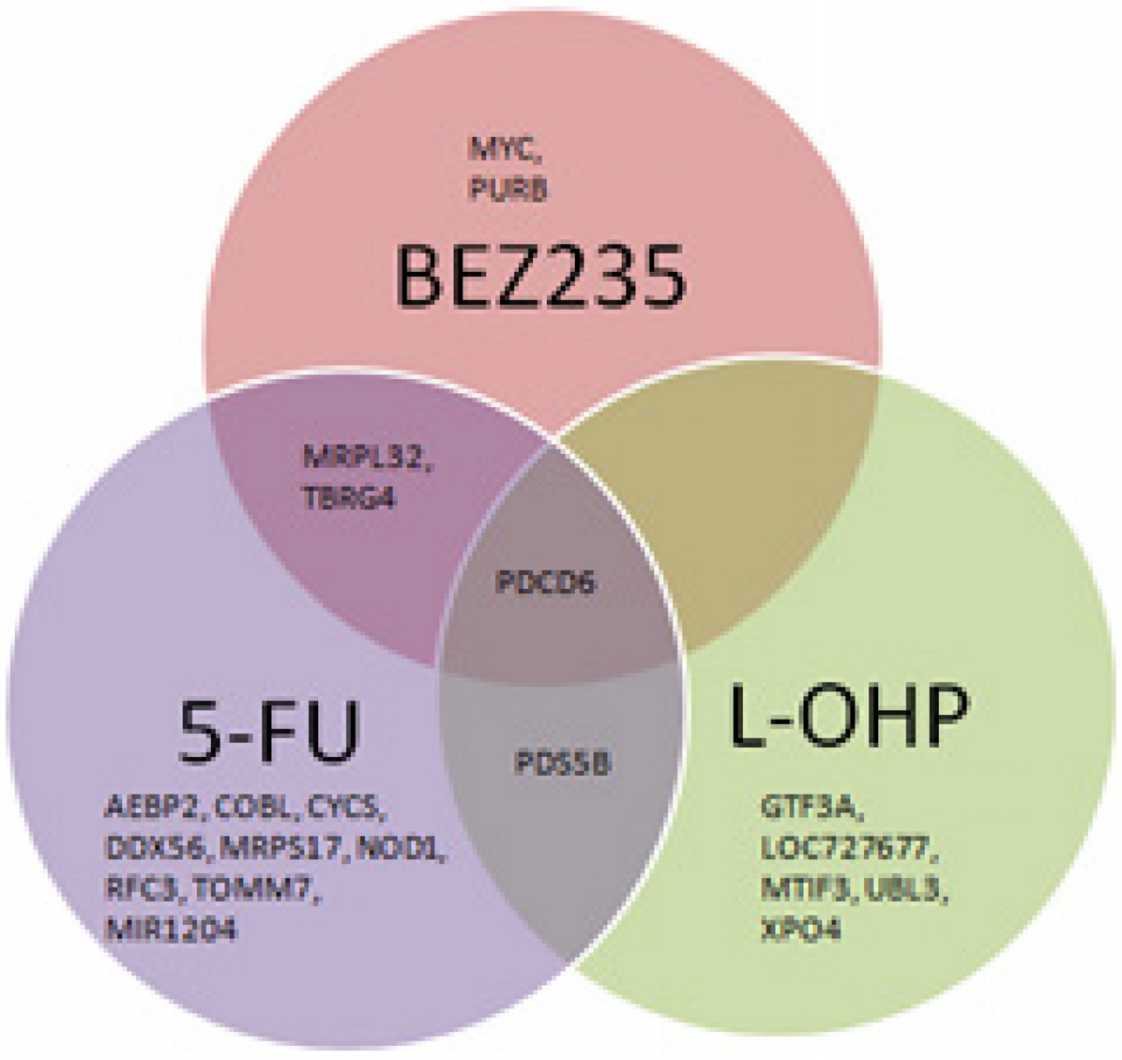
Venn diagram showing differentially expressed genes with respect to treatment response to a) 5-FU, b) L-OHP, and c) BEZ235B.

**Fig 6:**
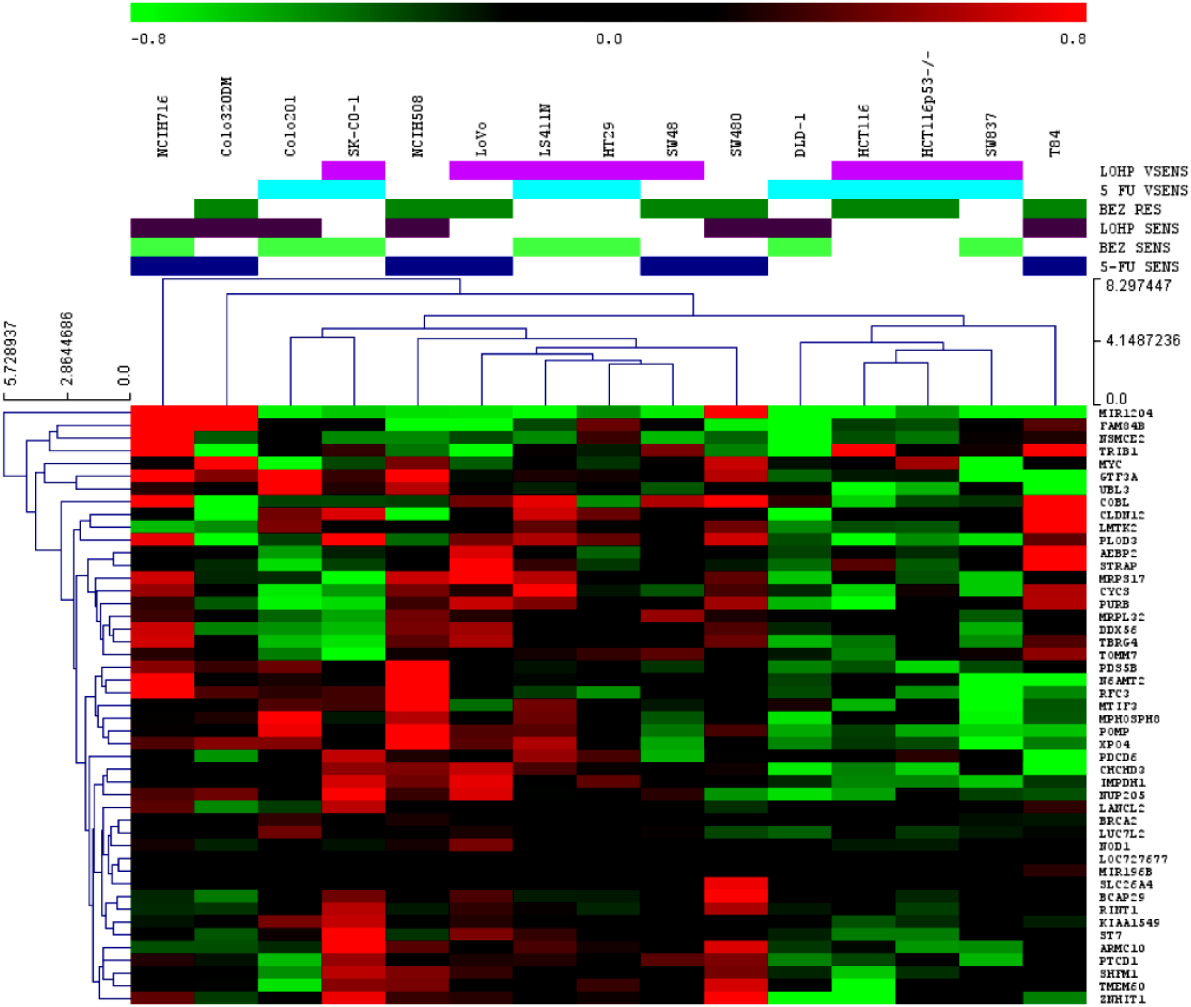
Unsupervised hierarchical clustering for the 47 candidate genes annotated according to response to therapy.

20/47 of these genes were associated with treatment responses (Figs 5 and 6). Significant differences were found between response to 5-FU treatment and gene expression of *TBRG4* (p ≤ 0.001), *MRPL32* (p ≤ 0.001), *CYCS* (p ≤ 0.001), *PDCD6* (p = 0.01), *COBL* (p = 0.01), *DDX56* (p = 0.01), *MRPS17* (p = 0.01), *PDS5B* (p = 0.03), *TOMM7* (p = 0.03), *AEBP2* (p = 0.04), *NOD1* (p = 0.04), *MIR1204* (p = 0.04) and *RFC3* (p = 0.05). Significant differences were found between response to BEZ235 treatment and gene expression of *PDCD6* (p = 0.002), *MYC* (p = 0.01), *MRPL32* (p = 0.01), *TBRG4* (p = 0.03) and *PURB* (p = 0.04). Significant differences were found between response to L-OHP treatment and gene expression of *PDS5B* (p < 0.005), *UBL3* (p = 0.01), *MTIF3* (p = 0.02), *CASC8* (p = 0.02), *XPO4* (p = 0.04), *GTF3A* (p = 0.04) and *PDCD6* (p = 0.04).

### Proteomic analysis

**Fig 7:**
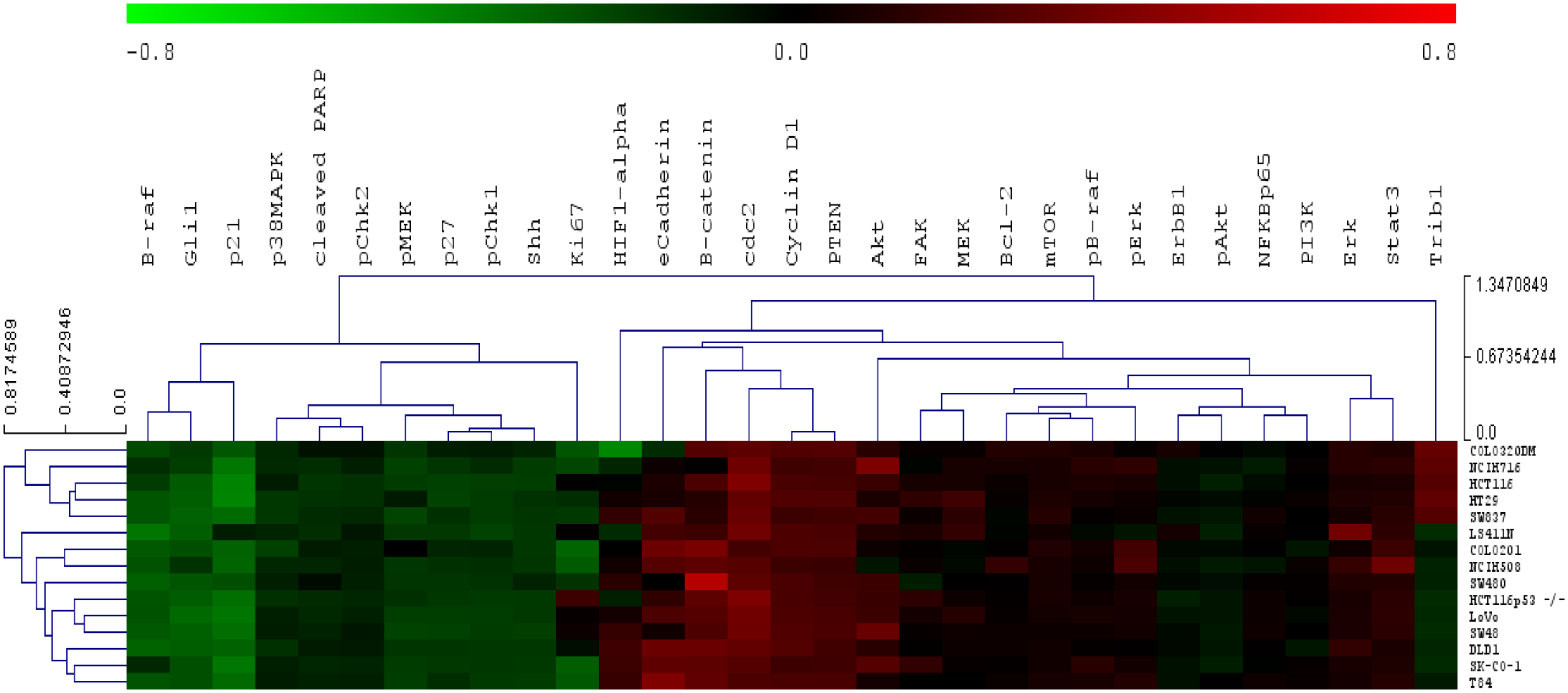
Unsupervised hierarchical clustering of RPPA protein expression data using Euclidian distance with average linkage.

Reverse phase protein array (RPPA) was used to measure protein expression of 31 phosphorylated and non-phosphorylated proteins in the CRC cell lines. Two main sub-clusters were produced by unsupervised hierarchical clustering of RPPA data (Fig 7). Sub-cluster one was enriched in proteins regulating cell-cycle function (Chk1, Chk2, p38MAPK, p21, p27, and Ki67; p = 0.037). Sub-cluster two was enriched for proteins regulating cell migration (Bcl-2, ErbB1, HIF-1 alpha, PTEN, TRIB1; p = 0.0007), phosphorylation (Bcl-2, cyclin D1, ErbB1, mTOR, PTEN, TRIB1; p = 0.001), cell proliferation (Bcl-2, β-catenin, cyclin D1, ErbB1, HIF-1 alpha, mTOR, PTEN, TRIB1; p = 0.0003), cellular responses to stress (Bcl-2, cdc2, cyclin D1, HIF-1 alpha, TRIB1; p = 0.001), negative regulation of apoptosis (Bcl-2, cdc2, ErbB1, PTEN, B-raf; p = 0.005), and focal adhesion (β-catenin, Bcl-2, B-Raf, cyclin D1, ErbB1, PTEN; p = 0.00013).

### Difference in protein expression with respect to treatment responses

**Fig 8:**
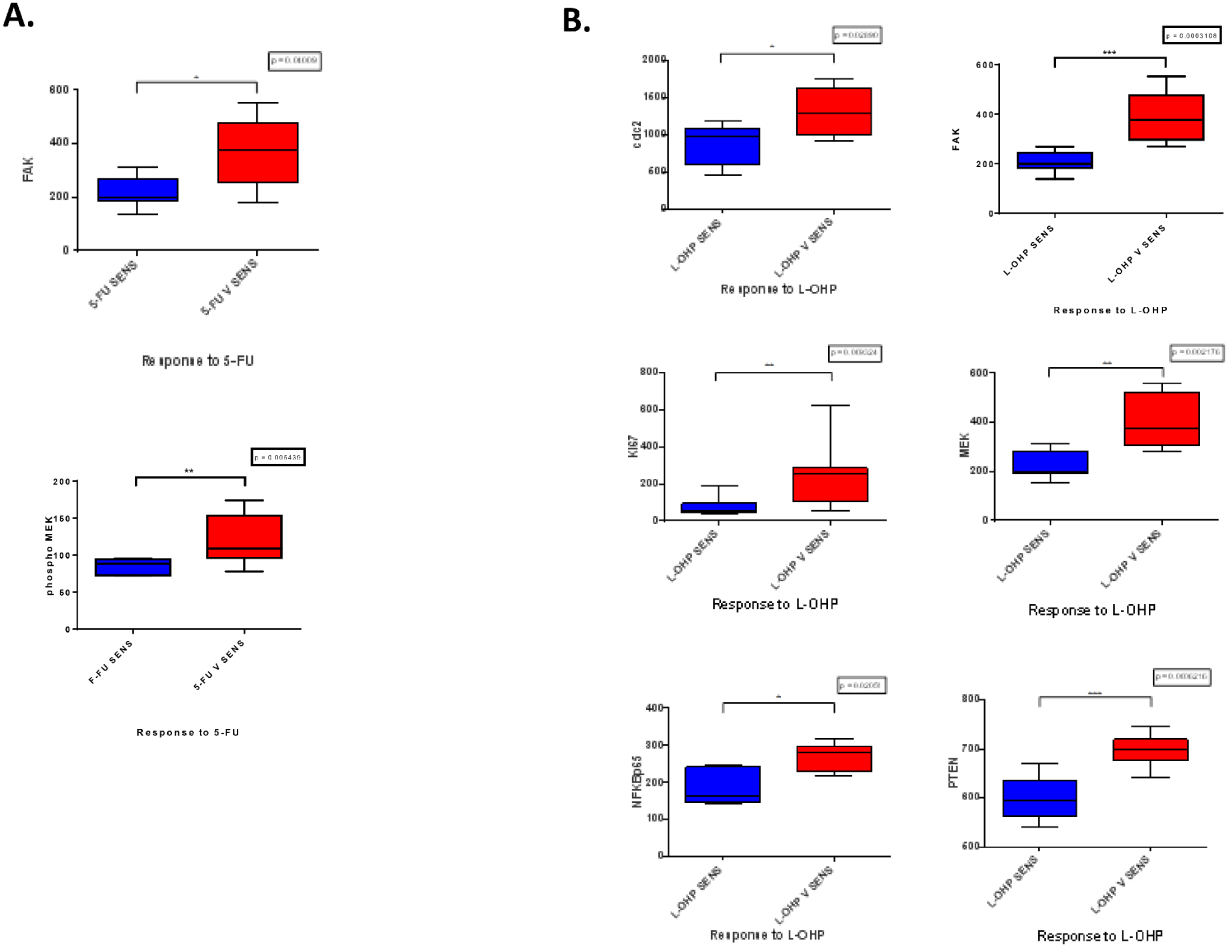
A. Box plots showing significant differences in protein expression between 5-FU sensitive and less sensitive cell lines (Mann Whitney U test) B. Box plots showing significant differences in protein expression between L-OHP sensitive and less sensitive cell lines (Mann Whitney U test).

Significant differences in protein expression were found for FAK (p = 0.004) and phospho MEK (p = 0.005) with respect to 5-FU treatment responses (Fig 8A). Significant differences in gene expression were found for cdc2 (p = 0.03), FAK (p = 0.0003), Ki67 (p = 0.009), MEK (p = 0.002), NFκβp65 (p = 0.02), and PTEN (p = 0.0006) with respect to L-OHP treatment responses (Fig 8B). No significant differences were observed for RPPA values with respect to response to BEZ235.

### *TRIB1* in CRC

Statistically significant correlations between copy number gains and gene expression were identified on amplicons located on chromosome 8. Candidate genes that could be investigated further included *TRIB1*, which was also observed to be recurrently amplified and overexpressed in a CRC study carried out by Camps et al. [41]. Furthermore, an integrated analysis of genomic and transcriptomic profiles of a panel of breast cancer cell lines established that TRIB1 is a potential amplicon driver [49]. TRIB1 has also been implicated as a key oncogene in acute myeloid leukaemia and ovarian cancers [50]. This region is 2.25Mb away from MYC, a well-established oncogene, including in CRC. TRIB1 was chosen as a candidate gene for further investigation due to the fact that seven out of fifteen cell lines exhibited copy number gain. The gene is located at Chr8: 126,393,571–126,567,050, in the 8q24 region, known to be associated with breast, ovarian, prostate and colorectal cancer [51]. TRIB1 is reported to be amplified in two integrated genomics and transcriptomic profiling studies on CRC cell lines and breast cancer cell lines, whereas in the latter TRIB1 was highlighted as a potential additional amplicon driver [41, 49]. Furthermore, the tribbles protein family act as adaptors that interact with the MAPK pathway [52], one of the most critical for cellular proliferation [53], transformation, differentiation [54], apoptosis, autophagic type II programmed cell death, and senescence [55]. In view of this pathway being centrally involved in cellular decision-making, small quantitative differences in pathway components may be sufficient to cause large changes in cellular phenotype [56].

### Genomic, transcriptomic, and proteomic data for *TRIB1* in the CRC cell line panel

There was a weak correlation between DNA copy number of the *TRIB1* region (Chr8: 126,393,571–126,567,050) and mRNA expression of *TRIB1* (r^2^ = 0.395, p = 0.012). The *TRIB1* region was gained in seven cell lines and clearly amplified and very highly expressed in NCI H716 cells. Reverse phase protein array (RPPA) analysis of TRIB1 was carried for the cell lines, which did not reveal a correlation with log2 copy number ratio (r^2^ = 0.209, p = 0.09) or with gene expression (r^2^ = 0.089, p = 0.282). Nevertheless, a large variation between TRIB1 protein expression was observed between the different cell lines that did not reach statistical significance.

### *TRIB1* and *MYC* amplification in the clinical cohort using FISH

The Oncomine^(R)^ [57] database was interrogated to explore *TRIB1* copy number in a cohort of 881 CRC patients (TCGA Colorectal 2), where *TRIB1* was found to be gained in 11% of primary CRC samples. Consequently, the amplification of *TRIB1* and *MYC* in the tissue microarray consisting of 118 Dukes’ A and B CRC patients was analysed.

Of the 118 cores (each representing a case), a total of 76 cores contained nuclei that could be scored for *TRIB1*. FISH scores for *TRIB1* ranged between 0.45 and 3.38 (median 1.00, IQR 0.28; mean 1.21, SD 0.52). Of 76 cases, 11 tumors (14.4%) were amplified (a score of ≥1.8).

Of 118 cores, a total of 81 cores contained nuclei that could be scored for *MYC*. FISH scores for *MYC* ranged between 0.70 and 4.14 (median 1.02; IQR 0.24; mean 1.17, SD 0.52). Six tumors were amplified for *MYC* (7.4%).

*TRIB1* and *MYC* FISH scores were strongly positively correlated (Spearman’s Rank; r^2^ = 0.783, p = 0.0001).

### *TRIB1* protein expression using AQUA and associated pathway expression

TRIB1 protein expression was next investigated using AQUA. Protein expression in the cytoplasm and nucleus was successfully measured in 96 out of 118 cases. Five samples out of the 96 samples showed TRIB1 overexpression (5.2%) in the cytoplasm when considering a cut-off of two standard deviations, while 6/96 showed TRIB1 overexpression (6.25%) in the nucleus.

**Fig 9:**
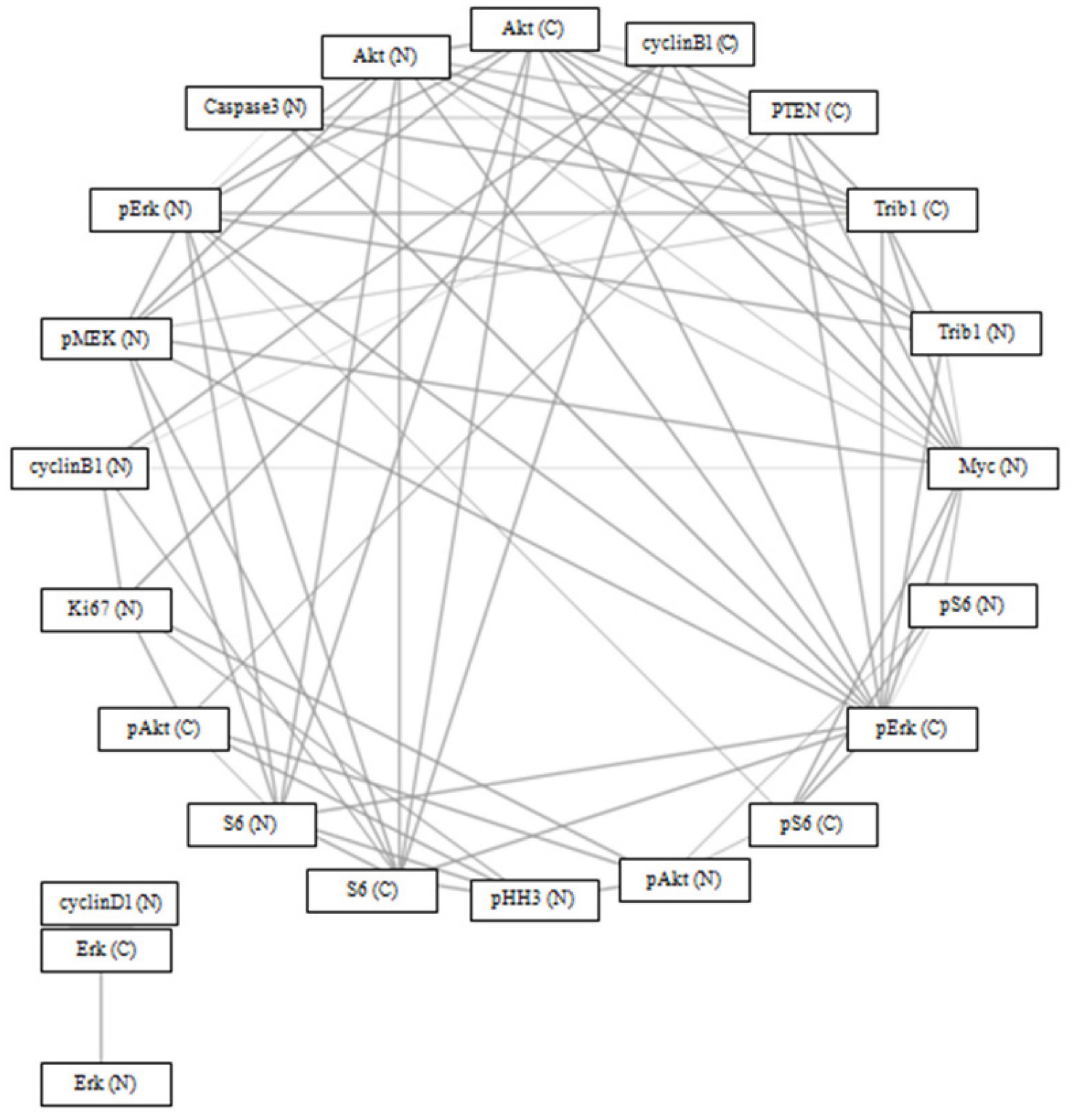
Spearman’s correlation network using Bonferroni Correction (p = 0.05) and circular network layout (http://www.tmanavigator.org/). Abbreviations: N—nucleus, C—cytoplasm.

Of 22 proteins in the MAPK pathway, TRIB1 protein expression in the cytoplasm was significantly correlated (p = 0.05) with TRIB1 (nucleus), phospho-Erk, Akt, Myc (nucleus), PTEN (cytoplasm), cleaved caspase 3 (nucleus), and phospho-MEK (nucleus), after correcting for multiple testing. TRIB1 protein expression in the nucleus was significantly correlated (p = 0.05) with TRIB1 (cytoplasm), Akt, phospho-Erk, and Myc (nucleus), after correcting for multiple testing (Fig 9). There was no statistically significant difference in survival between patients with *TRIB1* or *MYC* amplifications and those without.

## Discussion

Although the mutation status of a number of individual candidate genes has been associated with responses to CRC therapy, the results are inconclusive and few have resulted in useful stratification biomarkers. Here, the cellular response to treatment with 5-FU, L-OHP and BEZ235 was not associated with the mutational status of common genes in multiple cell lines. The measurement of a mutation in a single gene alone was insufficient to stratify patients for CRC therapy, which argues for adopting a multi-scale approach to help identify other factors that contribute to therapeutic resistance.

The list of tumor suppressor genes found in this study’s frequently deleted regions included *BCL2*, *DCC*, *CTDP1*, *SMAD2*, and *FHIT* [58–60]. Although *BCL2* is not usually considered to be a tumor suppressor gene, it has been reported to act as one under certain circumstances [61]. Furthermore, one of the frequently deleted regions contained *MACROD2* at 20p12.1 which was also described in a recently published study by Linnebacher et al. [62].

Systematic analysis of copy number gains allowed us to identify regions that were gained in seven or more cell lines. The use of a high-resolution array allowed analysis of frequently amplified regions that contained less well described genes. This analysis, when combined with gene and protein expression analysis and extensive literature review, helped us to identify a number of genes that could be further investigated as possible novel oncogenic drivers and determinants of response to therapy.

A number of genes were amplified, overexpressed, and associated with therapeutic responses. By adopting a functional multiscale analytical approach, a list of 20 candidate predictive biomarkers for 5-FU, L-OHP, and BEZ235 was generated. 5-FU-sensitive cell lines had higher programmed cell death 6 (*PDCD6*) gene expression than less sensitive cell lines. *PDCD6*, located on cytoband 5p15.33-p14.1, is known to be involved in apoptosis survival [63] and is implicated in migration and invasion in ovarian cancers [64]. Furthermore, there was a statistically significant difference with respect to treatment responses for the three treatments examined in this study. It has recently been demonstrated that *PDCD6* accumulates in the nucleus and induces apoptosis in response to DNA damage [65]. Moreover, Rho and colleagues found that over-expressed *PDCD6* inhibits angiogenesis through the PI3K/mToR/p70S6K pathway by interacting with VEGFR-2 [66], while Park et al. showed that PDCD6 exerts its anti-tumor potency by activating the p53-p21 protein for G_1_ phase of cell cycle progression and apoptosis involved in human ovarian tumorigenesis. This study suggested that suppressing *PDCD6* supports tumorigenesis by inhibiting apoptosis in ovarian cancer [67].

Expression of *TBRG4*, *MRPL32*, *CYCS*, *COBL*, *DDX56*, *MRPS17*, *PDS5B*, *TOMM7*, *AEBP2*, *NOD1*, *MIR1204* and *RFC3* was lower 5-FU-sensitive cell lines. *CYCS*, *TOMM7*, *NOD1*, *MRPL32*, *DDX56*, *TBRG4*, *COBL*, and *MRPS17* all map to the 7p21.1—7p11.2 cytoband. Their biological functions include positive regulation of cell proliferation and cell cycle arrest [68]. The Nod1 signalling complex has been shown to drive JNK activation, cytokine release, and induction of apoptosis in MCF7 breast cancer cells [69]. 7p21.1—7p11.2 cytoband amplification may in itself be, associated 5-FU responses by chromosomal-scale changes biasing expression over a large region and affecting genes that do not confer selective advantage [70]. Moreover, *EGFR* maps to this cytoband.

*PDS5B* has been shown to modulate homologous recombination in breast cancer and influence responses to DNA damaging agents [71]. Furthermore, they speculated that low *PDS5B*-expressing tumors are more responsive to DNA damaging chemotherapy [71]. *RFC3* copy number gains are frequently found in colon and oesophageal cancers, and in the latter cancer, Lockwood and colleagues showed that *RFC3* knockdown inhibited proliferation and anchorage-independent growth [72]. Furthermore, *RFC3* gene expression was one of the most differentially expressed between normal and tumor tissue [73]. *RFC3* is also involved in DNA synthesis and repair [74]. *MiR1204*, located on chromosome 8q24, may be associated with tumor growth suppression [75, 76], perhaps in a partially p53-dependent manner [77]. *AEBP2* is involved in DNA binding [78].

*PDCD6* gene expression was higher in cells sensitive to L-OHP, while expression of *PDS5B*, *UBL3*, *MTIF3*, *XPO4*, *CASC8*, *and GTF3A* was lower. *UBL3* was identified as one of seven genes that predict relapse and survival in early-stage cervical carcinoma patients [79]. *XPO4*, a critical protein synthesis regulator, is implicated in the regulation of Smad signalling [80].

*PDCD6* gene expression was, once again, greater in BEZ235-sensitive cell lines, while *MYC*, *MRPL32*, *TBRG4*, *and PURB* was lower. The frequent association of PDCD6 gene expression with drug response supports future studies to explore the significance of this gene with respect to drug response. A number of *in vitro* and *in vivo* studies in breast and prostate cancer have demonstrated that *MYC* amplification or phosphorylation lead to acquired resistance to BEZ235 [81], and Tan and colleagues used a PDK1 inhibitor to bypass *MYC*-dependent resistance [81]. Genomic amplification of *MYC* or *eIF4E* contributed to resistance to BEZ235 in mammary epithelial cells [82]. *MRPL32*, *TBRG4*, and *PURB* all mapped to chromosome 7p14.2-p11.2. Chromosome 7p gains have been observed in both the early- and late-stage CRC [83]. *TBRG4* is involved in positive regulation of cell proliferation and cell cycle arrest [68] and apoptosis [84].

Seven proteins were associated with responses to cytotoxic therapies, but no differential expression was seen with the PI3K/mTOR inhibitor. For example, focal adhesion kinase (FAK) was differentially expressed between 5-FU and L-OHP groups. FAK is associated with apoptosis and proliferation pathways in cancer cell lines [85]. Cdc2 was similarly differentially expressed between L-OHP very sensitive and less sensitive cell lines. *CDK1* (which codes for cdc2) loss elicited chemotherapeutic resistance in lung cancer [86], while cdc2 was differentially expressed in a study of responses to L-OHP three CRC cell lines [87].

As proof of concept of adopting a functional multi-scale analytical approach to comprehend the underlying changes driving colorectal carcinogenesis, a gene that was frequently amplified, *TRIB1*, was selected for further analysis as a candidate biomarker. There was a highly statistically significant correlation between the FISH score of *TRIB1* and *MYC* (r^2^ = 0.783, p = 0.0001), consistent with co-amplification. A number of studies have suggested that *MYC*-driven cancers are reliant on other genes and pathways, unlike non-*MYC*-driven cancers [88–90]. Toyoshima and colleagues identified a set of 102 genes required for survival of c-MYC over-expressing cells using a high-throughput siRNA screening approach (91), which included *TRIB1*. Furthermore, *TRIB1* appears to be druggable, involved in oncogenic pathways, and differential toxicity. Gene expression silencing of *TRIB1* using deconvoluted siRNA pool-mediated knockdown resulted in increase in cleaved caspase 3 and 7 and in increase of γ-H2AX foci in c-MYC expressing human foreskin fibroblasts but not in the control fibroblasts [91].

We speculate that *MYC* and *TRIB1* are co-amplified in a number of CRC patients and that targeting *TRIB1* would lead to cell death via a synthetic lethal mechanism. Since *MYC* cannot be therapeutically targeted, it would be useful to investigate the function of *TRIB1* and its role in targeted therapy. *MYC* is known to interact with a number of signalling pathways and is mostly involved in growth and proliferation. Furthermore, although *MYC* is prominently referred to as a proto-oncogene, *MYC* also exhibits pro-apoptotic properties [92]. It is feasible that in a subset of CRCs, when *TRIB1* is targeted, *MYC* might function as a tumor suppressor gene leading to cell death. This would need to be validated in a series of functional experiments.

In addition, TRIB1 protein expression was significantly correlated with MYC, phosphorylated MEK, ERK, total Akt, PTEN, and cleaved caspase 3, consistent with previous findings that TRIB1 interacts with MEK1 and overexpression leads to ERK phosphorylation [93]. Furthermore, a number of studies have observed that TRIB1 is predominantly, but not exclusively, located in the nucleus, as here [52]. Both TRIB2 and TRIB3 have interact with Akt, mainly by inhibition, but no data has yet been published for TRIB1 [94]. These data must be interpreted with caution but it would be interesting to investigate the involvement of TRIB1 in the MAPK and PI3K/Akt pathway.

Although a difference in TRIB1 expression was observed in the cell line panel, there was no statistically significant correlation between gene and protein expression. This could have occurred for a number of technical, statistical, and biological reasons including assay sensitivity, array probe specificity, mRNA and protein degradation [95], and sample numbers. Sharova and colleagues confirmed that TRIB1 has an mRNA half-life of less than one hour, in spite of the median estimated half-life being 7.1 hours [96]. This finding sheds some light on the functional role of TRIB1, in that the half-life is related to its physiological role and usually found in transcription factors and genes involved in cell cycling [97]. Additionally, a number of transcripts encoding regulatory proteins are known to undergo rapid mRNA decay [98].

This study has a number of limitations. Further data analysis needs to be performed with respect to frequently deleted regions to identify putative tumor suppressor genes involved in CRC and their relationship with treatment responses. This study used continuous cancer cell lines, which may not fully represent parent tumors and, therefore, clinical responses to therapy. Nevertheless, cell lines have been shown to recapitulate the molecular and phenotypic characteristics of primary tumors [99–101], including in colorectal cancer [102], and therefore have value in translational studies and biomarker discovery. Finally, the tumors analysed in the clinical cohort were derived from patients less than 50 years of age and might not be fully representative of the wider CRC population. Further validation is required in a larger, more representative clinical cohort.

## Conclusions

Our multi-scale analytical approach has generated a list of 20 candidate predictive biomarkers for 5-FU, L-OHP, and BEZ235. This approach is valuable for understanding the mode of action of different treatments and guiding personalised therapy. Furthermore, we show, for the first time, that *TRIB1* is co-amplified with *MYC* in a proportion of CRCs and may be an attractive target for intervention in this group of patients.

## Supporting Information

S1 Table Patient characteristics of the study population (n = 118). (DOCX)

S2 Table Antibodies used in the RPPA analysis. (DOCX)

S3 Table Differentially expressed genes for 5-FU response of the 15 CRC cell lines having a 1.5-fold change or more. (DOCX)

S4 Table Differentially expressed genes for L-OHP response of the 15 CRC cell lines having at least a 1.5-fold change. (DOCX)

S5 Table Differentially expressed genes for BEZ235 response of the 15 CRC. (DOCX)
