# Supplementary material for: Multi-Scale Genomic, Transcriptomic and Proteomic Analysis of Colorectal Cancer Cell Lines to Identify Novel Biomarkers
Source: PLoS One. 2015 Dec 17;10(12):e0144708. doi: 10.1371/journal.pone.0144708 (PMC4692059; doi:10.1371/journal.pone.0144708)
Supplement: S1 Table — (DOCX) [file pone.0144708.s001.docx]

**S1 Table.** Patient characteristics of the study population (n=118)

| Sex | Male  Female |  | 59 (50)  59 (50) |
| --- | --- | --- | --- |
| Age |  |  | 27 – 55, median = 48 |
| Location | Caecum  Ascending Colon/Hepatic Flexure  Transverse Colon  Descending Colon  Sigmoid  Rectosigmoid  Rectum  Hepatic flexure  Splenic Flexure  Appendix |  | 11 (9.32)  14 (11.86)  2 (1.69)  7 (5.93)  27 (22.88)  14 (11.86)  35 (29.66)  3 (2.54)  4 (3.39)  1 (0.85) |
| Differentiation | Well  Moderate  Poor  N/A |  | 7 (5.93)  92 (77.97)  13 (11.02)  6 (5.08) |
| Histology | Standard  Mucinous  Neuroendocrine  N/A |  | 90 (76.27)  24 (20.34)  2 (1.69)  2 (1.69) |
| Staging | Dukes’ A  Dukes’ B  Dukes’ C |  | 31 (26.27)  83 (70.34)  4 (3.39) |
