# Supplementary material for: Multi-Scale Genomic, Transcriptomic and Proteomic Analysis of Colorectal Cancer Cell Lines to Identify Novel Biomarkers
Source: PLoS One. 2015 Dec 17;10(12):e0144708. doi: 10.1371/journal.pone.0144708 (PMC4692059; doi:10.1371/journal.pone.0144708)
Supplement: S2 Table — (DOCX) [file pone.0144708.s002.docx]

**S2 Table.** Antibodies used in the RPPA analysis

| **Antibody Target** | **Host** | **Manufacturer** | **Cat. No.** | **Dilution** |
| --- | --- | --- | --- | --- |
| Akt | Mouse | Cell Signaling | 2920 | 1:1000 |
| Bcl-2 | Rabbit | Eurogentech | 75380 | 1:50 |
| B-Raf | Mouse | Santa-Cruz | sc-5284 | 1:150 |
| CDK1 | Rabbit | Eurogentech | 75557 | 1:50 |
| Cleaved PARP (Asp214) | Mouse | Cell Signaling | 9546 | 1:50 |
| Cyclin D1 | Mouse | Cell Signaling | 2926 | 1:300 |
| E-Cadherin | Mouse | BD | 610181 | 1:50 |
| EGFR | Mouse | Cell Signaling | 2239 | 1:125 |
| ERK | Mouse | Cell Signaling | 9107 | 1:50 |
| FAK | Rabbit | Cell Signaling | 3285 | 1:100 |
| GLI1 | Mouse | Cell Signaling | 2643 | 1:50 |
| Hif-1α | Rabbit | Cell Signaling | 3716 | 1:50 |
| Ki67 | Mouse | DAKO | M7240 | 1:100 |
| MEK ½ | Mouse | Cell Signaling | 4694 | 1:50 |
| mTOR | Rabbit | Cell Signaling | 2972 | 1:300 |
| NFKβ p65 | Rabbit | Cell Signaling | 4764 | 1:50 |
| p21 Waf1 Cip1 | Mouse | Cell Signaling | 2946 | 1:150 |
| p27 Kip1 | Mouse | Cell Signaling | 3698 | 1:50 |
| p38 MAPK | Rabbit | Cell Signaling | 9212 | 1:100 |
| phospho-Akt (Ser473) | Rabbit | Cell Signaling | 9271 | 1:50 |
| phospho-B-Raf (Ser445) | Rabbit | Cell Signaling | 2696 | 1:150 |
| phospho-Chk1 (Ser317) | Rabbit | Cell Signaling | 2344 | 1:200 |
| phospho-Chk2 (Ser516) | Rabbit | Cell Signaling | 2669 | 1:50 |
| phospho-MEK1/2 (Ser217/221) | Rabbit | Cell Signaling | 9154 | 1:50 |
| phospho-p44/42 MAPK (Erk1/2) (Thr202/Tyr204) | Rabbit | Cell Signaling | 9101 | 1:100 |
| PI3K p110α | Rabbit | Cell Signaling | 4249 | 1:133 |
| PTEN | Mouse | Cell Signaling | 9556 | 1:300 |
| Shh | Rabbit | Cell Signaling | 2207 | 1:50 |
| STAT3 | Rabbit | Eurogentech | 75367 | 1:50 |
| TRIB-1 | Rabbit | Abcam | ab78214 | 1:50 |
| β-catenin | Mouse | BD | 610153 | 1:50 |
