# Supplementary material for: Multi-Scale Genomic, Transcriptomic and Proteomic Analysis of Colorectal Cancer Cell Lines to Identify Novel Biomarkers
Source: PLoS One. 2015 Dec 17;10(12):e0144708. doi: 10.1371/journal.pone.0144708 (PMC4692059; doi:10.1371/journal.pone.0144708)
Supplement: S3 Table — (DOCX) [file pone.0144708.s003.docx]

**S3 Table.** Differentially expressed genes for 5-FU response of the 15 CRC cell lines having a 1.5-fold change or more.

| **Identifier** | **Description** | **PC (gene vs. outcome):** | **Fold Change** | **Q-value (Rank)** |
| --- | --- | --- | --- | --- |
| ANXA3 | annexin A3 | 0.62 | 3.66 | 0 (65.) |
| MLPH | Melanophilin | 0.64 | 3.35 | 0 (41.) |
| SCNN1A | sodium channel, nonvoltage-gated 1 alpha | 0.63 | 3.33 | 0 (47.) |
| SLC16A5 | solute carrier family 16, member 5 (monocarboxylic acid transporter 6) | 0.7 | 2.75 | 0 (12.) |
| PROM2 | prominin 2 | 0.68 | 2.58 | 0 (21.) |
| GSTO2 | glutathione S-transferase omega 2 | 0.6 | 2.49 | 0 (89.) |
| SH3KBP1 | SH3-domain kinase binding protein 1 | 0.67 | 2.34 | 0 (28.) |
| TMEM125 | transmembrane protein 125 | 0.61 | 2.33 | 0 (84.) |
| SLC45A3 | solute carrier family 45, member 3 | 0.64 | 2.26 | 0 (48.) |
| HYOU1 | hypoxia up-regulated 1 | 0.63 | 2.17 | 0 (59.) |
| NXN | Nucleoredoxin | 0.61 | 1.98 | 0 (90.) |
| LMCD1 | LIM and cysteine-rich domains 1 | 0.65 | 1.95 | 0 (42.) |
| ZNF239 | zinc finger protein 239 | 0.69 | 1.93 | 0 (17.) |
| CHURC1 | farnesyltransferase, CAAX box, beta | 0.62 | 1.92 | 0 (72.) |
| EFR3A | EFR3 homolog A (S. cerevisiae) | 0.71 | 1.85 | 0 (11.) |
| GSDMD | gasdermin D | 0.66 | 1.84 | 0 (34.) |
| TATDN1 | TatD DNase domain containing 1 | 0.78 | 1.82 | 0 (1.) |
| TRAM1 | translocation associated membrane protein 1 | 0.71 | 1.8 | 0 (8.) |
| HOOK2 | hook homolog 2 (Drosophila) | 0.69 | 1.78 | 0 (18.) |
| TSTD1 | thiosulfate sulfurtransferase (rhodanese)-like domain containing 1 | 0.68 | 1.76 | 0 (27.) |
| FER1L4 | Unknown | 0.6 | 1.75 | 0 (98.) |
| LOC374395 | Unknown | 0.67 | 1.72 | 0 (30.) |
| ADAM9 | ADAM metallopeptidase domain 9 | 0.64 | 1.72 | 0 (49.) |
| WDR41 | WD repeat domain 41 | 0.64 | 1.72 | 0 (53.) |
| CDK2AP2 | cyclin-dependent kinase 2 associated protein 2 | 0.68 | 1.65 | 0 (29.) |
| OSGIN2 | oxidative stress induced growth inhibitor family member 2 | 0.68 | 1.64 | 0 (26.) |
| COMT | catechol-O-methyltransferase | 0.61 | 1.64 | 0 (93.) |
| LACTB2 | lactamase, beta 2 | 0.61 | 1.63 | 0 (85.) |
| PLEKHA1 | pleckstrin homology domain containing, family A (phosphoinositide binding specific) member 1 | 0.72 | 1.62 | 0 (6.) |
| NOXO1 | NADPH oxidase organizer 1 | 0.71 | 1.62 | 0 (13.) |
| FTSJD1 | FtsJ methyltransferase domain containing 1 | 0.65 | 1.62 | 0 (40.) |
| OBFC1 | oligonucleotide/oligosaccharide-binding fold containing 1 | 0.63 | 1.6 | 0 (63.) |
| HSP90B1 | heat shock protein 90kDa beta (Grp94), member 1 | 0.65 | 1.58 | 0 (45.) |
| C8orf76 | chromosome 8 open reading frame 76 | 0.75 | 1.57 | 0 (3.) |
| LOC341315 | Unknown | 0.64 | 1.57 | 0 (54.) |
| VPS28 | vacuolar protein sorting 28 homolog (S. cerevisiae) | 0.65 | 1.56 | 0 (43.) |
| STT3A | STT3, subunit of the oligosaccharyltransferase complex, homolog A (S. cerevisiae) | 0.63 | 1.56 | 0 (64.) |
| GPR172A | G protein-coupled receptor 172A | 0.64 | 1.55 | 0 (55.) |
| CYB561 | cytochrome b-561 | 0.61 | 1.55 | 0 (88.) |
| NT5C2 | 5'-nucleotidase, cytosolic II | 0.71 | 1.52 | 0 (15.) |
| TERF1 | telomeric repeat binding factor (NIMA-interacting) 1 | 0.69 | 1.52 | 0 (22.) |
| MTFR1 | mitochondrial fission regulator 1 | 0.64 | 1.52 | 0 (52.) |
| EBAG9 | estrogen receptor binding site associated, antigen, 9 | 0.71 | 1.49 | 0 (14.) |
| TSTA3 | tissue specific transplantation antigen P35B | 0.69 | 1.49 | 0 (25.) |
| DNAL4 | dynein, axonemal, light chain 4 | 0.63 | 1.48 | 0 (70.) |
| CHRAC1 | chromatin accessibility complex 1 | 0.74 | 1.47 | 0 (4.) |
| TSPAN14 | tetraspanin 14 | 0.63 | 1.47 | 0 (66.) |
| TRIOBP | TRIO and F-actin binding protein | 0.62 | 1.47 | 0 (91.) |
| FAM149B1 | family with sequence similarity 149, member B1 | 0.63 | 1.46 | 0 (76.) |
| PXMP3 | Unknown | 0.77 | 1.45 | 0 (2.) |
