# Supplementary material for: Multi-Scale Genomic, Transcriptomic and Proteomic Analysis of Colorectal Cancer Cell Lines to Identify Novel Biomarkers
Source: PLoS One. 2015 Dec 17;10(12):e0144708. doi: 10.1371/journal.pone.0144708 (PMC4692059; doi:10.1371/journal.pone.0144708)
Supplement: S4 Table — (DOCX) [file pone.0144708.s004.docx]

**S4 Table.** Differentially expressed genes for L-OHP response of

the 15 CRC cell lines having at least a 1.5-fold change.

| **Identifier** | **Description** | **PC (gene vs. outcome):** | **Fold Change** | **Q-value (Rank)** |
| --- | --- | --- | --- | --- |
| *CMBL* | carboxymethylenebutenolidase homolog (Pseudomonas) | 0.63 | 3.36 | 0 (45.) |
| *KCNK1* | potassium channel, subfamily K, member 1 | 0.73 | 3.28 | 0 (4.) |
| *GSTO2* | glutathione S-transferase omega 2 | 0.74 | 3.05 | 0 (3.) |
| *KRTCAP3* | keratinocyte associated protein 3 | 0.64 | 2.76 | 0 (40.) |
| *SNHG8* | Unknown | 0.67 | 2.24 | 0 (21.) |
| *MTAP* | methylthioadenosine phosphorylase | 0.69 | 2.23 | 0 (13.) |
| *EPHB4* | EPH receptor B4 | 0.67 | 2.15 | 0 (24.) |
| *CCNO* | cyclin O | 0.65 | 2.14 | 0 (35.) |
| *STK39* | serine threonine kinase 39 | 0.7 | 2.06 | 0 (11.) |
| *TNFSF9* | tumor necrosis factor (ligand) superfamily, member 9 | 0.6 | 2.03 | 0.91 (95.) |
| *CBLC* | Cas-Br-M (murine) ecotropic retroviral transforming sequence c | 0.63 | 1.82 | 0 (59.) |
| *LOC642989* | Unknown | 0.7 | 1.81 | 0 (12.) |
| *LOC387825* | Unknown | 0.71 | 1.76 | 0 (9.) |
| *MOSC2* | MOCO sulphurase C-terminal domain containing 2 | 0.64 | 1.75 | 0 (53.) |
| *MAP4K2* | mitogen-activated protein kinase kinase kinase kinase 2 | 0.69 | 1.7 | 0 (18.) |
| *RPS27L* | ribosomal protein S27-like | 0.61 | 1.67 | 0.91 (88.) |
| *NRBP2* | nuclear receptor binding protein 2 | 0.64 | 1.65 | 0 (51.) |
| *PAQR7* | progestin and adipoQ receptor family member VII | 0.65 | 1.63 | 0 (43.) |
| *LOC387791* | Unknown | 0.76 | 1.58 | 0 (2.) |
| *GLI2* | GLI family zinc finger 2 | 0.62 | 1.5 | 0 (73.) |
| *NFXL1* | nuclear transcription factor, X-box binding-like 1 | 0.67 | 1.49 | 0 (27.) |
| *RPL13A* | ribosomal protein L13a | 0.62 | 1.49 | 0.91 (79.) |
| *GRTP1* | growth hormone regulated TBC protein 1 | 0.66 | 1.48 | 0 (41.) |
| *EML3* | echinoderm microtubule associated protein like 3 | 0.68 | 1.47 | 0 (22.) |
| *EHD2* | EH-domain containing 2 | 0.64 | 1.47 | 0 (63.) |
| *XPNPEP1* | X-prolyl aminopeptidase (aminopeptidase P) 1, soluble | 0.7 | 1.46 | 0 (15.) |
| *GNB1L* | guanine nucleotide binding protein (G protein), beta polypeptide 1-like | 0.65 | 1.46 | 0 (46.) |
| *SPRYD3* | SPRY domain containing 3 | 0.61 | 1.46 | 0.91 (92.) |
| *CRLF3* | cytokine receptor-like factor 3 | 0.62 | 1.45 | 0 (74.) |
