# Supplementary material for: Multi-Scale Genomic, Transcriptomic and Proteomic Analysis of Colorectal Cancer Cell Lines to Identify Novel Biomarkers
Source: PLoS One. 2015 Dec 17;10(12):e0144708. doi: 10.1371/journal.pone.0144708 (PMC4692059; doi:10.1371/journal.pone.0144708)
Supplement: S5 Table — (DOCX) [file pone.0144708.s005.docx]

**S5 Table**. Differentially expressed genes for BEZ235 response of the

15 CRC Cell lines having at least a 1.5-fold change.

| ***Identifier*** | **Description** | **PC (gene vs. outcome):** | **Fold Change** | **Q-value (Rank)** |
| --- | --- | --- | --- | --- |
| *TFF3* | trefoil factor 3 (intestinal) | 0.62 | 11.56 | 0 (43.) |
| *LYZ* | Lysozyme | 0.66 | 8.15 | 0 (22.) |
| *C9orf152* | chromosome 9 open reading frame 152 | 0.67 | 4.18 | 0 (19.) |
| *MLPH* | Melanophilin | 0.75 | 4.12 | 0 (1.) |
| *C1orf59* | chromosome 1 open reading frame 59 | 0.68 | 3.48 | 0 (16.) |
| *RPESP* | Unknown | 0.59 | 3.13 | 0 (99.) |
| *CTSH* | cathepsin H | 0.64 | 3.1 | 0 (33.) |
| *SNTB1* | syntrophin, beta 1 (dystrophin-associated protein A1, 59kDa, basic component 1) | 0.67 | 2.97 | 0 (18.) |
| *MUC5AC* | mucin 5AC, oligomeric mucus/gel-forming | 0.68 | 2.87 | 0 (14.) |
| *RAP1GAP* | RAP1 GTPase activating protein | 0.6 | 2.72 | 0 (80.) |
| *PRTFDC1* | phosphoribosyl transferase domain containing 1 | 0.66 | 2.43 | 0 (26.) |
| *LIMCH1* | LIM and calponin homology domains 1 | 0.69 | 2.36 | 0 (11.) |
| *PROM2* | prominin 2 | 0.6 | 2.31 | 0 (86.) |
| *UCP2* | uncoupling protein 2 (mitochondrial, proton carrier) | 0.64 | 2.27 | 0 (35.) |
| *CHURC1* | farnesyltransferase, CAAX box, beta | 0.69 | 2.07 | 0 (9.) |
| *MSRB2* | methionine sulfoxide reductase B2 | 0.61 | 2.04 | 0 (72.) |
| *SPRY1* | sprouty homolog 1, antagonist of FGF signaling (Drosophila) | 0.65 | 2 | 0 (36.) |
| *C5orf39* | chromosome 5 open reading frame 39 | 0.6 | 2 | 0 (89.) |
| *LMCD1* | LIM and cysteine-rich domains 1 | 0.65 | 1.96 | 0 (31.) |
| *G6PD* | glucose-6-phosphate dehydrogenase | 0.62 | 1.94 | 0 (55.) |
| *ANXA9* | annexin A9 | 0.62 | 1.92 | 0 (57.) |
| *C4orf34* | chromosome 4 open reading frame 34 | 0.63 | 1.91 | 0 (48.) |
| *ARSD* | arylsulfatase D | 0.68 | 1.89 | 0 (15.) |
| *SPDEF* | SAM pointed domain containing ets transcription factor | 0.63 | 1.87 | 0 (49.) |
| *ECH1* | enoyl CoA hydratase 1, peroxisomal | 0.73 | 1.82 | 0 (2.) |
| *PROX1* | prospero homeobox 1 | 0.63 | 1.82 | 0 (44.) |
| *TIMP4* | TIMP metallopeptidase inhibitor 4 | 0.64 | 1.8 | 0 (42.) |
| *SEC24D* | SEC24 family, member D (S. cerevisiae) | 0.61 | 1.8 | 0 (70.) |
| *MGLL* | monoglyceride lipase | 0.62 | 1.74 | 0 (65.) |
| *KLHDC2* | kelch domain containing 2 | 0.63 | 1.69 | 0 (52.) |
| *HINT2* | histidine triad nucleotide binding protein 2 | 0.67 | 1.68 | 0 (21.) |
| *PIK3C2B* | phosphoinositide-3-kinase, class 2, beta polypeptide | 0.6 | 1.67 | 0 (92.) |
| *SCARB2* | scavenger receptor class B, member 2 | 0.62 | 1.6 | 0 (66.) |
| *SH3TC1* | SH3 domain and tetratricopeptide repeats 1 | 0.61 | 1.6 | 0 (81.) |
| *SSR4* | signal sequence receptor, delta (translocon-associated protein delta) | 0.6 | 1.58 | 0 (100.) |
| *LOC644914* | Unknown | 0.71 | 1.56 | 0 (6.) |
| *RAB11A* | RAB11A, member RAS oncogene family | 0.62 | 1.56 | 0 (68.) |
| *LOC730740* | Unknown | 0.69 | 1.55 | 0 (17.) |
| *PPT1* | palmitoyl-protein thioesterase 1 | 0.61 | 1.55 | 0 (84.) |
| *ERN2* | endoplasmic reticulum to nucleus signaling 2 | 0.62 | 1.54 | 0 (78.) |
| *LRP5* | low density lipoprotein receptor-related protein 5 | 0.73 | 1.53 | 0 (3.) |
| *LOC347376* | Unknown | 0.65 | 1.52 | 0 (37.) |
| *H3F3A* | H3 histone, family 3A | 0.66 | 1.49 | 0 (30.) |
| *NUCB1* | nucleobindin 1 | 0.63 | 1.48 | 0 (61.) |
| *CD63* | CD63 molecule | 0.61 | 1.48 | 0 (82.) |
| *CHMP4A* | chromatin modifying protein 4A | 0.72 | 1.47 | 0 (4.) |
